# Metadata analysis to explore hub of the hub-genes highlighting their functions, pathways and regulators for cervical cancer diagnosis and therapies

**DOI:** 10.1007/s12672-022-00546-6

**Published:** 2022-08-22

**Authors:** Md. Selim Reza, Md. Alim Hossen, Md. Harun-Or-Roshid, Mst. Ayesha Siddika, Md. Hadiul Kabir, Md. Nurul Haque Mollah

**Affiliations:** 1 Bioinformatics Lab, Department of Statistics, University of Rajshahi, Rajshahi-6205, Bangladesh; 2 Microbiology Lab, Department of Veterinary and Animal Sciences, University of Rajshahi, Rajshahi-6205, Bangladesh

**Keywords:** Cervical cancer (CC), Hub genes (HubGs), Hub of the HubGs (hHubGs), Candidate drugs, Integrated bioinformatics analysis

## Abstract

Cervical cancer (CC) is considered as the fourth most common women cancer globally.that shows malignant features of local infiltration and invasion into adjacent organs and tissues. There are several individual studies in the literature that explored CC-causing hub-genes (HubGs), however, we observed that their results are not so consistent. Therefore, the main objective of this study was to explore hub of the HubGs (hHubGs) that might be more representative CC-causing HubGs compare to the single study based HubGs. We reviewed 52 published articles and found 255 HubGs/studied-genes in total. Among them, we selected 10 HubGs (CDK1, CDK2, CHEK1, MKI67, TOP2A, BRCA1, PLK1, CCNA2, CCNB1, TYMS) as the hHubGs by the protein–protein interaction (PPI) network analysis. Then, we validated their differential expression patterns between CC and control samples through the GPEA database. The enrichment analysis of HubGs revealed some crucial CC-causing biological processes (BPs), molecular functions (MFs) and cellular components (CCs) by involving hHubGs. The gene regulatory network (GRN) analysis identified four TFs proteins and three miRNAs as the key transcriptional and post-transcriptional regulators of hHubGs. Then, we identified hHubGs-guided top-ranked FDA-approved 10 candidate drugs and validated them against the state-of-the-arts independent receptors by molecular docking analysis. Finally, we investigated the binding stability of the top-ranked three candidate drugs (Docetaxel, Temsirolimus, Paclitaxel) by using 100 ns MD-based MM-PBSA simulations and observed their stable performance. Therefore the finding of this study might be the useful resources for CC diagnosis and therapies.

**Supplementary Information:**

The online version contains supplementary material available at 10.1007/s12672-022-00546-6.

## Introduction

Cancer has the largest clinical, social, and economic impact in terms of cause-specific disability-adjusted life years [1]. Cervical cancer (CC) is the fourth most common cancer in women and has the fourth highest mortality rate globally [2]. The most common causes of cancer-related mortality among individuals with CC are invasion and metastasis by CC cells, which are associated with a poor prognosis [3, 4]. Fortunately, effective primary and secondary preventive methods, including as human papillomavirus (HPV) vaccine and yearly cytology smears, are available for CC. Almost all CC patients have a long-term infection with high-risk HPV types [5–7]. For early-stage and low-risk CC, surgery, chemotherapy, or radiation have shown to be effective [8–10]. However, metastatic cervical cancer (mCC) has a 5-year survival rate of 16.5 percent [11]. Additionally, the efficacy of chemotherapy and radiation is reduced by their side effects. Therefore, exploration of biological causes as well as the development of new treatment targets and techniques for CC are required to increase patient survival.

The identification of biomarkers that might possibly alleviate the disorder's pathogenesis could be a motivating factor that leads to the development of more effective therapy options in the future [12]. The potential benefits of molecular biomarkers suggest that they might improve the efficacy of CC diagnostic and treatment. Bioinformatics techniques are now widely used in several of biological research areas. Recently, sequencing tools have become more widely available, allowing researchers to make significant findings in both computational biology and molecular therapies [13]. Protein–Protein Interactions (PPI) were previously utilized to identify the hub genes that may be responsible for the disease, and a co-expression network was employed to validate the listed genes using a heat map based on their co-regulation scores [14–17]. Protein 3D structures are important for the fields in evolutionary biology and biotechnology, such as protein function prediction and drug design [18].

**Fig. 1:**
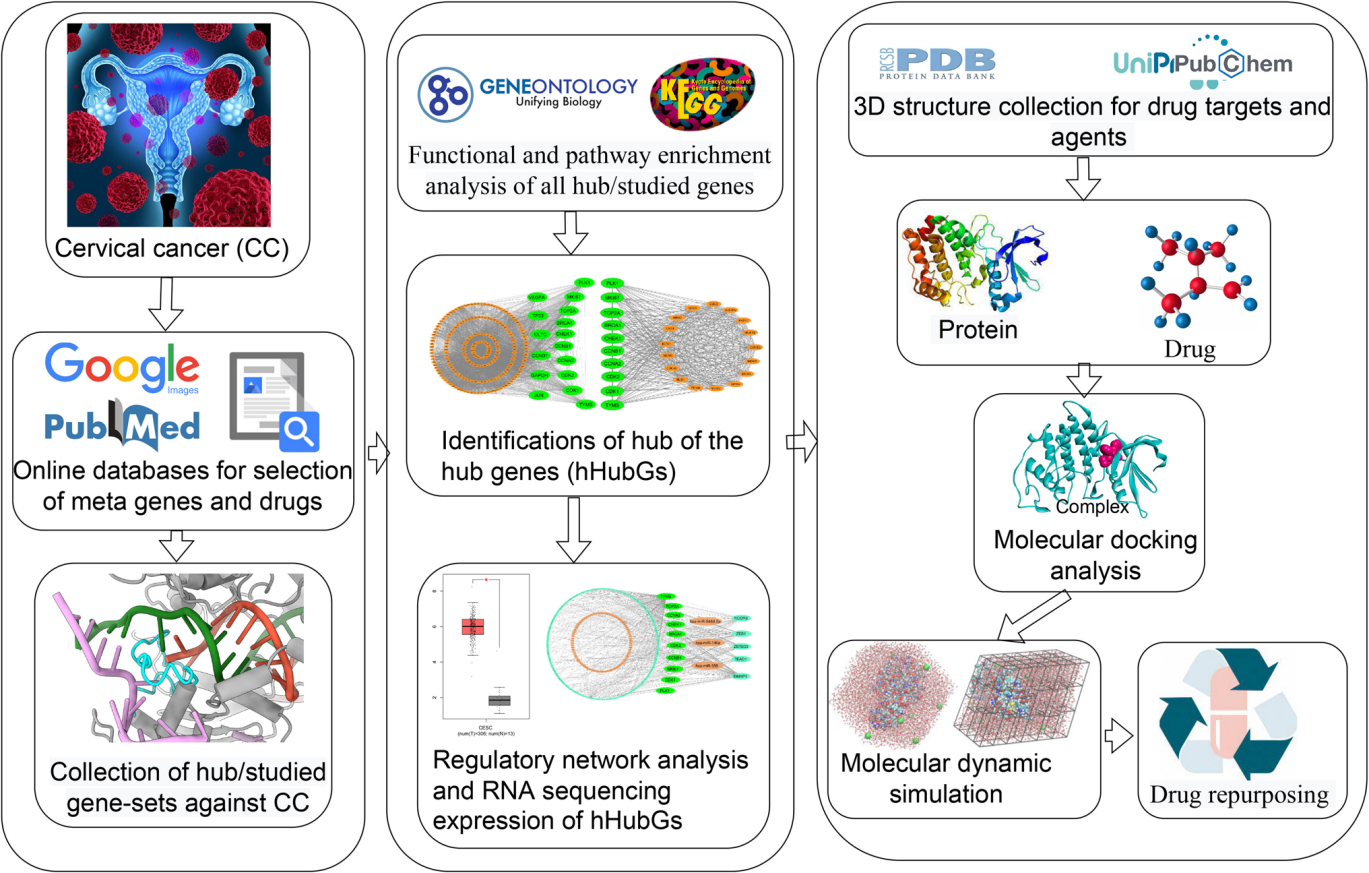
The workflow of this study

However, new drug development is a difficult, time-consuming, and costly endeavor. The major challenges are to identify disease-causing drug target proteins and drug agents that can alleviate disease severity by interacting with the target proteins. As opposed to developing a new drug, repurposing existing drugs for certain conditions might save time and money. By this time, numerous studies have proposed different sets of hub/key-genes to explore molecular mechanisms and pathogenetic processes in CC [19–24]. Some of these studies have also been suggested their hub-genes or studied-gene guided candidate drugs for the treatment of CC [23, 24]. By the literature review, we observed that their suggested CC-causing hub-genes (HubGs) or studied-genes sets as well as candidate drugs are not so consistent in different published articles. On the other hand, so far, none of these studies investigated the performance of their suggested drugs against the other published hub-genes mediated target proteins. Obviously, more representative hub-genes are required to explore more effective candidate drugs against CC. Therefore, in this study, our main objectives are to (1) explore hub of the HubGs (hHubGs) highlighting their functions, pathways, and regulatory factors, (2) explore hHubGs-guided candidate drugs for the treatment against CC, and (3) cross-validation of the proposed candidate drugs against the state-of-the-arts alternatives independent target proteins published by others. The workflow of this study is demonstrated in Fig. 1.

## Materials and methods

In this study, the necessary meta-drug target and agents were collected from different online sources and published articles to explore globally most effective repurposable drugs for the treatment against CC by using the integrated bioinformatics approaches.

### Metadata sources and descriptions

We have collected metadata for both drug targets and agents associated with CC to reach the goal of this study as described below.

### Collection of studied/hub-DEGs to explore drug targets

Several research groups already published different sets of studied or hub differentially expressed genes (studied/hub-DEGs) associated with CC [12, 13, 20, 22–71]. Here DEGs indicates the differentially expressed genes between CC and control samples. The present study included the articles in 2010–2021 associated with CC infections.To select the top-ranked hub-genes (independent meta-receptors**)** associated with CC disease; we reviewed 52 published articles and collected 255 hub meta-receptors to explore key genes (KGs) by protein–protein interaction (PPI) network analysis [see Table S1]. The proteins corresponding to our proposed KGs would be considered as the key drug targets.

### Collection of drug agents

Several research groups already suggested transcriptome-guided different sets of repurposable drugs for the treatment against CC [23, 24, 69, 70, 72–91]. We collected host transcriptome-guided 80 meta-drug agents by the literature review of CC disease [see Table S2] for exploring candidate drugs.

### Protein–protein interaction (PPI) network analysis of all Hub/Studied-DEGs

The STRING online database (https://string-db.org/) was used to construct the PPI network of DEGs. [92]. We utilized the Cytoscape software to enhance the quality of the PPI network [93]. To select common Hub-Genes (cHubGs) or common Hub-Proteins (cHubPs) from the PPI network, the Cytoscape plugin cytoHubba was implemented [93, 94]. The PPI network is comprised of a number of nodes and edges that represent proteins and their interactions, respectively. A node with the largest number of significant interactions/connections/edges with other nodes is considered the top-ranked cHubGs. Four topological analyses of PPI network were applied to select the cHubGs (Degree [95], BottleNeck [96], Betweenness [97], and Stress [98]). The Cytoscape software's Molecular Complex Detection (MCODE) (http://apps.cytoscape.org/apps/mcode) plugin was used to identify the most significant modules in the PPIs network. MCODE clustering detected highly interconnected portions, which helps the study with effective drug design. By identifying densely connected regions, MCODE was utilized to represent molecular complexes in the PPIs network [99]. The hub of the hub-genes (hHubGs) were then selected since they were shared by both cHubGs and MCODE clustering genes.

### Differential expression patterns analysis of hHubGs

To validate the expression of the hHubGs, the Gene Expression Profiling Interactive Analysis (GEPIA) website was applied to analyze the data of RNA sequencing expression based on thousands of samples from the GTEx projects and TCGA.

### GO terms and KEGG pathway enrichment analysis of all Hub/Studied-DEGs

Gene ontology (GO) functional and Kyoto encyclopedia of genes and genomes (KEGG) pathway enrichment/annotation/over-representation analysis [100, 101] is a widely used approach to determine the significantly annotated/enriched/over-represented functions/classes/terms and pathways by the identified KGs. It is indeed vital for understanding how genes work at the molecular level and what role they play in the cell. Biological Process, Cellular Component, and Molecular Function are the three categories of GO terms [102]. We used the DAVID online tool (https://david.ncifcrf.gov/tools.jsp) to perform GO and KEGG enrichment analysis [103]. The p-value 0.05 was selected as the significance level.

### Regulatory network analysis of hHubGs

Using the NetworkAnalyst online tool, we performed TFs-hHubGs and miRNAs-hHubGs interaction network analysis on hHubGs to uncover major transcriptional regulatory transcription factors (TFs) and post-transcriptional regulatory micro-RNAs (miRNAs) [104]. The ENCODE (https://www.encodeproject.org/) [105] and RegNetwork repository [106] databases were used to construct the TFs- hHubGs and miRNAs- hHubGs interaction networks, respectively. To enhance the quality of the networks, the Cytoscape software [93] was utilized. Then, we determined the regulators (TFs or miRNAs) based on their high connectivity with hHubGs.

### Drug repurposing by molecular docking study

We performed a molecular docking analysis of our suggested receptor proteins with drug agents to propose in-silico validated efficient candidate drugs for the treatment of CC. As previously mentioned in the data sources (see Table S1), we considered our proposed hHubGs based key proteins (KPs) and their regulatory key TFs proteins as drug target proteins and 77 meta-drug agents. Both receptor proteins and meta-drug agents require 3-Dimensional (3D) structures for molecular docking studies. All of the targeted proteins' 3D structures were downloaded from the Protein Data Bank (PDB) [107] and SWISS-MODEL [108]. All meta-drug agents' 3D structures were downloaded from the PubChem database [109]. Using discovery studio visualizer 2019 [110], the 3D structures of the target proteins were displayed, and target chains that were not part of the gene were deleted. The protonation state of protein was assigned using the PDB2PQR and H +  + servers [111, 112]. As well, all absent hydrogen atoms were properly added. The pKa for the receptor amino acids were examined under the physical conditions of pH = 7, salinity = 0.15, external dielectric = 80, and internal dielectric = 10. Then, using AutoDock tools, the receptor was prepared for molecular docking study by eliminating water molecules, and ligand heteroatoms and by addition of polar hydrogens [113]. The grid box was generated over the entire surface of the proteins. The ligands were prepared for molecular docking study by using AutoDock tools to set the torsion tree and rotatable and nonrotatable bonds in the ligand. AutoDock Vina was used to calculate binding affinities between target proteins and drug agents [114]. The exhaustiveness parameter was set to 10. PyMol [115] and discovery studio visualizer 2019 [110] were used to analyse the docked complexes for surface complexes, types, and distances of non-covalent bonds. Let *A*_*ij*_ denotes the binding affinity between *i*^th^ target protein (*i* = 1, 2, …, *m*) and *j*^th^ drug agent (*j* = 1, 2, …, *n*). To select the top-ranked lead compounds as the candidate drugs, we ordered the drug target proteins and agents according to the descending order of row sums ${\sum }_{j=1}^{n}{A}_{ij}$, *j* = 1,2,…,*m*, and column sums ${\sum }_{i=1}^{m}{A}_{ij}$, *j* = 1,2,…,*n*, respectively.

### Molecular dynamic (MD) simulations

To discover the dynamic behavior of the top-ranked protein–ligand complexes, MD simulations were performed using YASARA Dynamics software [116], and the AMBER14 force field [117]. A total of six different systems were used to run MD simulation. The systems included Docetaxel-CDK1, Temsirolimus-CHEK1, Paclitaxel-TOP2A, apo-CDK1, apo-CHEK1, and apo-TOP2A.

For the complexes, the ligand parameters for the complexes were assigned using AutoSMILES [118] algorithms. A TIP3P [119] water model in a simulation cell was used for optimized and solvated the hydrogen bonding network of the protein–ligand complexes before the simulation. With a solvent density of 0.997 gL1, periodic boundary conditions were maintained. Titratable amino acids in the complexes were subjected to pKa calculation during solvation. The initial energy minimization process of each simulation system, consisting of of 55,410 ± 10, 72,287 ± 10, and 96,252 ± 10 atoms for CDK1_Docetaxel, CHEK1_Temsirolimus, and TOP2A_Paclitaxel complexes were performed by a simulated annealing method respectively, using the steepest gradient approach (5000 cycles). For the details of MD simulation methods see our previous paper [14, 15]. The trajectories were recorded every 250 ps for further analysis, and subsequent analysis was implemented by default script of YASARA [120] macro and SciDAVis software available at http://scidavis.sourceforge.net/. All snapshots were then subjected to YASARA software's MM-Poisson–Boltzmann surface area (MM-PBSA) binding free energy calculation using the formula below [121],

$$\mathrm{Binding free Energy}= {\mathrm{E}}_{\mathrm{potReceptor}}+{\mathrm{E}}_{\mathrm{solvReceptor}}+ {\mathrm{E}}_{\mathrm{potLigand}}+ {\mathrm{E}}_{\mathrm{solvLigand}}- {\mathrm{E}}_{\mathrm{potComplex}}- {\mathrm{E}}_{\mathrm{solvComplex}}$$

Here, MM-PBSA binding energy was calculated using YASARA built-in macros using AMBER 14 as a force field, with larger positive energies indicating better binding [122].

## Results

### Basic characteristics of the selected studies

The goal of this study was to identify the potential biomarkers and repurposing drug agents against cc. To screening the published articles, we conducted title, abstract, result, and conclusion with cervical cancer, hub genes/targets/receptors/proteins, number of drug agents/compounds/ligands, and collected 52 published articles from online databases to collect the hub genes or study genes. To select the top-ranked independent meta-receptors associated with CC disease, we gather 255 hub meta-receptors from 52 published articles to explore hub of the Hub-DEGs (hHubGs) by protein–protein interaction (PPI) network analysis [see Table S1]. On the other hand, we acquired 23 published papers from online databases to select the meta-drug agents/compounds/ligands against CC disease and accumulated host transcriptome-guided 77 meta-drug agents from 23 reputed published papers against CC disease [see Table S2] for exploring the candidate drugs.

### Identification of hub of hub-genes (hHubGs)

**Fig. 2:**
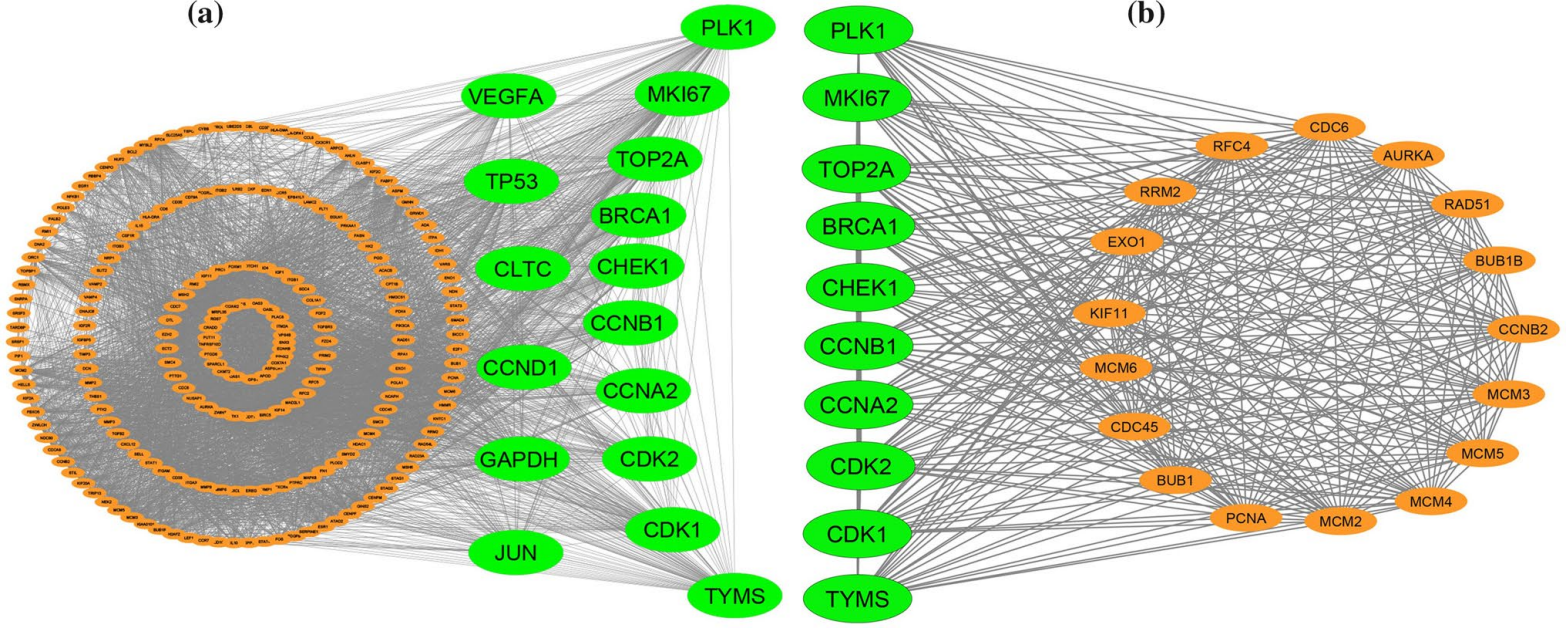
**a** Protein-proteins interaction network for common differentially expressed genes of CC, and edges specify the interconnection in the middle of two genes. The analyzed network holds 229 nodes and 4488 edges. Surrounding nodes (PLK1, TP53, GAPDH, VEGFA, CDK2, MKI67, CHEK1, CDK1, BRCA1, TOP2A, CLTC, CCNA2, JUN, CCND1, CCNB1, TYMS) represented the hub genes. **b** Module analysis network obtained from MCODE analysis. Surrounding nodes (CDK2, CHEK1, MKI67, TOP2A, CDK1, BRCA1, PLK1, CCNA2, CCNB1, TYMS) were found to be common across 10 hub genes, so we considered these ten genes as the key genes. The network represents highly interconnected regions of the PPIs network. The network holds 27 nodes and 350 edges

To identify the hHub-proteins, the protein protein interaction (PPI) network analysis was utilized by using the all collected hub-DEGs from the selected published papers. The PPI network of cDEGs was constructed using STRING database (Fig. 2a) which contains 229 nodes and 4488 edges. We selected top-ranked sixteen (16) cHubGs {PLK1, TP53, GAPDH, VEGFA, CDK2, MKI67, CHEK1, CDK1, BRCA1, TOP2A, CLTC, CCNA2, JUN, CCND1, CCNB1, TYMS} applying four topological measures in the PPI network. Then, using MCODE, clusters were selected from the PPI network. It was shown that the most significant cluster had 27 nodes and 350 edges (see Table S3). MCODE analysis demonstrated that the most significant cluster contained the ten hHubGs {CDK2, CHEK1, MKI67, TOP2A, CDK1, BRCA1, PLK1, CCNA2, CCNB1, TYMS} (see Fig. 2b). These top hHubGs may be focused for the pre-clinical potential drug target molecule that may open a new era of therapeutic targets.

### Differential expression patterns analysis of hHubGs

**Fig. 3:**
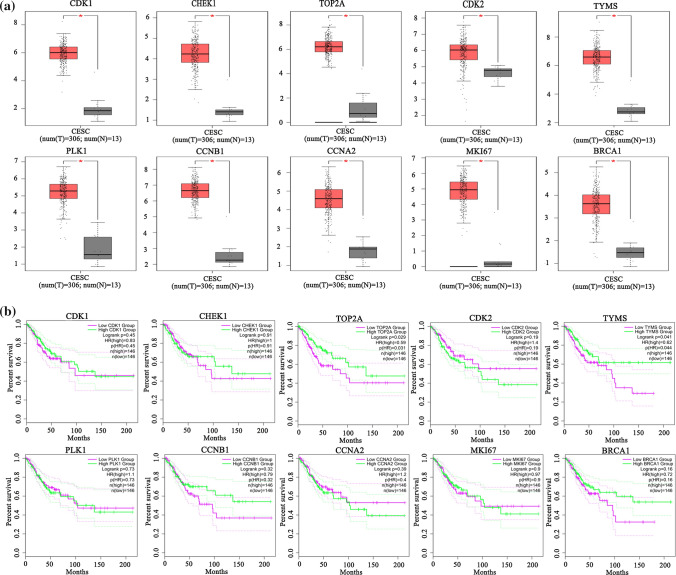
**a** Validation of the expression of 10 hHubGs in CC tissues via GEPIA website. Red color represents tumor samples, while gray color represents normal samples. **b** Overall survival of 10 hHubGs in CC patients. The green curve is the high expression group and the magenta curve is the low-expression group

To investigate the differential expression patterns of hHubGs (CDK2, CHEK1, MKI67, TOP2A, CDK1, BRCA1, PLK1, CCNA2, CCNB1, TYMS) between CC and control samples, we performed box-plot analysis by using the GEPIA web-tool (see Fig. 3a). We observed the all hHubGs were significantly differentially exprerssed between CC and control samples. On the other hand, The GEPIA database was also used to investigate the prognostic power of these 10 hHubGs in CC patients using by using their survival analysis. From Fig. 3b, we observed that the lower expressions of two hHubGs (CDK2 and CCNA2) and higher expression of (CHEK1, TOP2A, BRCA1, CCNB1 and TYMS) increases the survival probability of patients, significanty.

### Functional and pathway enrichment analysis of all Hub/Studied-DEGs

The GO functional enrichment analysis revealed that our proposed hHubGs significantly enriched with abundant number of biological processes (BPs), molecular functions (MFs) and cellular components (CCs) (Table 1 and Table S1). The Table 1 shows top 5 significantly enriched GO-terms for each of three categories (BPs, MFs, and CCs). These functions and pathways are highly connected with the CC related biological functional pathways in host which are crucial for developing therapeutic targets. The top five GO terms of the biological process including DNA replication, cell division, G1/S transition of mitotic cell cycle, mitotic nuclear division, and regulation of signal transduction by p53 class mediator were significantly enriched by the hHubGs-sets {BRCA1, CHEK1, CDK2, CDK1}, {CCNB2, CCNB1, CDK2, CDK1}, {TYMS, CDK2, CDK1}, {CCNB2, CDK2, CDK1}, and {BRCA1, CHEK1, CDK2}, respectively. The MFs GO terms protein binding, chromatin binding, ATP binding, protein kinase binding, and protein heterodimerization activity were significantly enriched by the hHubGs-sets {CHEK1, CCNB2, CCNB1, CDK2, CDK1}, {TOP2A, CDK1}, {TOP2A, CDK2, CDK1}, {CCNB1}, and {TOP2A}, respectively. The Cellular Components GO terms nucleoplasm, Cytosol, nucleus, spindle pole, and cytoplasm were significantly enriched by the hHubGs-sets {TOP2A, BRCA1, CHEK1, CCNB2, CCNB1, CDK2, CDK1}, {CHEK1, CCNB2, CCNB1, CDK2, CDK1}, {MKI67, CHEK1, CCNB2, CCNB1, CDK2, CDK1, TOP2A}, {CCNB1}, and {MKI67, CCNB1, CDK2, TOP2A, BRCA1}, respectively. We also observed that KEGG pathway categories Cell cycle, Pathways in cancer, HTLV-I infection, Hepatitis B, and p53 signaling pathway were significantly enriched by the hHubGs-sets {CCNB2, CCNB1, CHEK1, CDK2, CDK1}, {CDK2}, {CHEK1}, {CDK2}, and {CCNB2, CCNB1, CHEK1, CDK2, CDK1}, respectively. The other significantly enriched GO terms and KEGG pathways of hHubGs-sets were given in Table S4.

**Table 1:** The top five significantly (p-value < 0.001) enriched GO functions and KEGG pathways by hub/studied-genes involving hHubGs with CC diseases

Biological Process (BPs)				
GO ID	GO Term	Hub-DEGs (Counts)	P-Value	Associated hHubGs
GO:0,006,260	DNA replication	33	2.32E-28	BRCA1, CHEK1, CDK2, CDK1
GO:0,051,301	cell division	37	5.39E-21	CCNB2, CCNB1, CDK2, CDK1
GO:0,000,082	G1/S transition of mitotic cell cycle	22	6.39E-19	TYMS, CDK2, CDK1
GO:0,007,067	mitotic nuclear division	28	1.38E-16	CCNB2, CDK2, CDK1
GO:1,901,796	regulation of signal transduction by p53 class mediator	18	1.78E-12	BRCA1, CHEK1, CDK2
Molecular Function (MFs)				
GO ID	GO Term	Hub-DEGs (Counts)	P-Value	Associated hHubGs
GO:0,005,515	protein binding	192	1.39E-19	CHEK1, CCNB2, CCNB1, CDK2, CDK1
GO:0,003,682	chromatin binding	28	1.08E-11	TOP2A, CDK1
GO:0,005,524	ATP binding	55	8.87E-11	TOP2A, CDK2, CDK1
GO:0,019,901	protein kinase binding	22	9.59E-08	CCNB1
GO:0,046,982	protein heterodimerization activity	24	2.07E-07	TOP2A
Cellular Component				
GO ID	GO Term	Hub-DEGs (Counts)	P-Value	Associated hHubGs
GO:0,005,654	nucleoplasm	93	6.62E-19	TOP2A, BRCA1, CHEK1, CCNB2, CCNB1, CDK2, CDK1
GO:0,005,829	cytosol	91	5.86E-13	CHEK1, CCNB2, CCNB1, CDK2, CDK1
GO:0,005,634	nucleus	115	2.22E-09	MKI67, CHEK1, CCNB2, CCNB1, CDK2, CDK1, TOP2A
GO:0,000,922	spindle pole	13	1.91E-08	CCNB1
GO:0,005,737	cytoplasm	108	5.49E-08	MKI67, CCNB1, CDK2, TOP2A, BRCA1
KEGG Pathway				
has ID	Pathways	Hub-DEGs (Counts)	P-Value	Associated hHubGs
hsa04110	Cell cycle	30	6.63E-21	CCNB2, CCNB1, CHEK1, CDK2, CDK1
hsa05200	Pathways in cancer	39	1.88E-13	CDK2
hsa05166	HTLV-I infection	28	1.11E-10	CHEK1
hsa05161	Hepatitis B	20	2.47E-09	CDK2
hsa04115	p53 signaling pathway	11	5.32E-06	CCNB2, CCNB1, CHEK1, CDK2, CDK1

### Transcriptional and post transcriptional regulatory factors of hHubGs

**Fig. 4:**
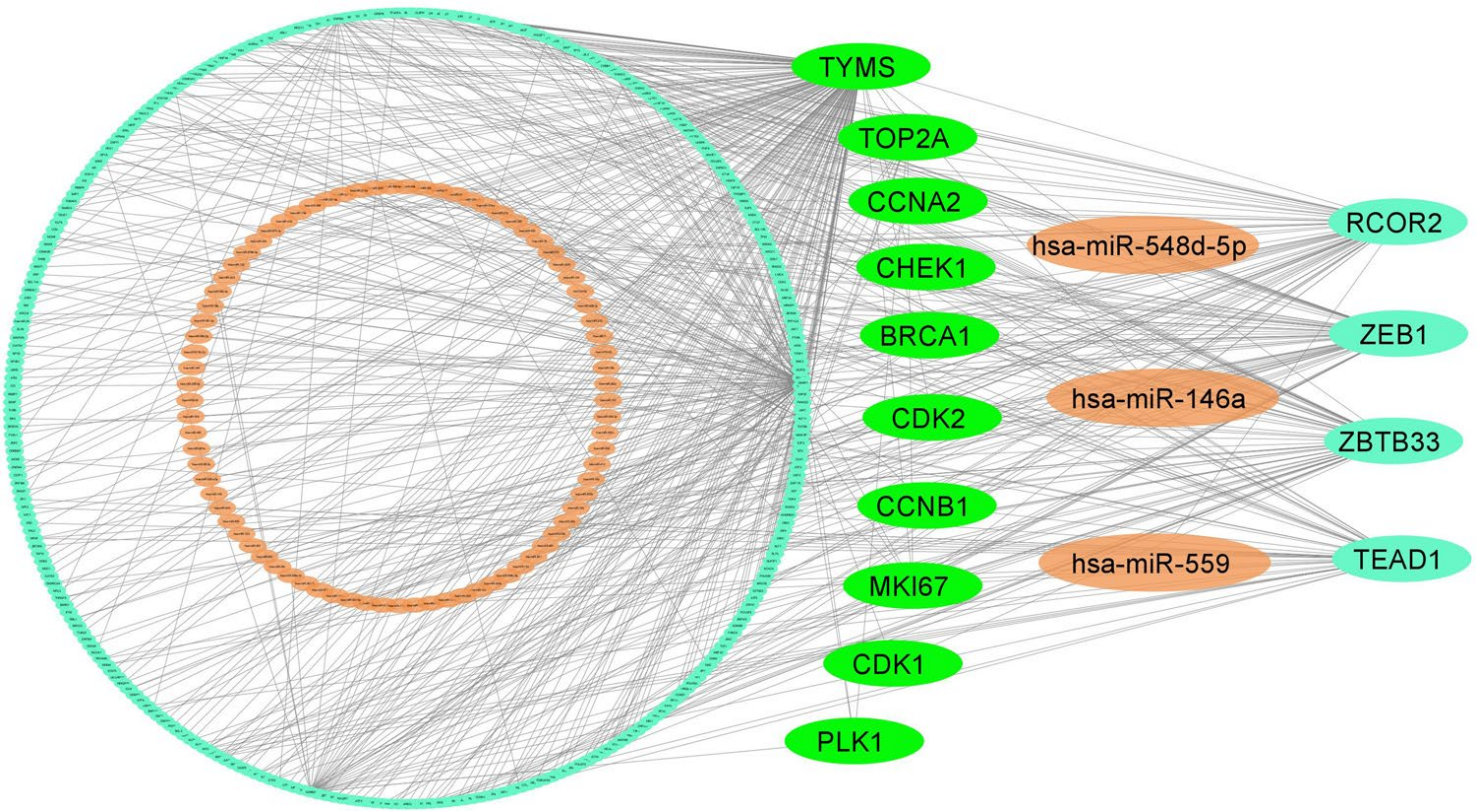
TFs-genes- miRNAs interaction network with hHubGs. The highlighted green color nodes represent the hHubGs, orange color nodes represent the miRNAs, and other cyan color nodes represent TFs. The network consists of 400 nodes and 583 edges

The network analysis of hHubGs with TFs detected top-ranked four significant TFs (TEAD1, ZBTB33, RCOR2, and ZEB1) as the key transcriptional regulatory factors for hHubGs (see Fig. 4). We found that TEAD1 is key TFs of three hHubGs (TYMS, CCNB1, and MKI67), ZBTB33 for four hHubGs (TYMS, CDK2, MKI67, and CDK1), RCOR2 for three hHubGs (TYMS, MKI67, and TOP2A), and ZEB1 for three hHubGs (BRCA1, CDK2, and MKI67). Similarly, the network analysis of hHubGs with miRNAs identified top-ranked three significant miRNAs denote as hsa-miR-548d-5p, hsa-miR-146a and hsa-miR-559 that are considered as the key post-transcriptional regulatory factors for hHubGs-sets {CCNA2, CDK2, BRCA1}, { CHEK1, TYMS, BRCA1, TOP2A}, and { CDK2, MKI67, CCNB1} respectively (see Fig. 4).

### Drug repurposing by molecular docking

**Fig. 5:**
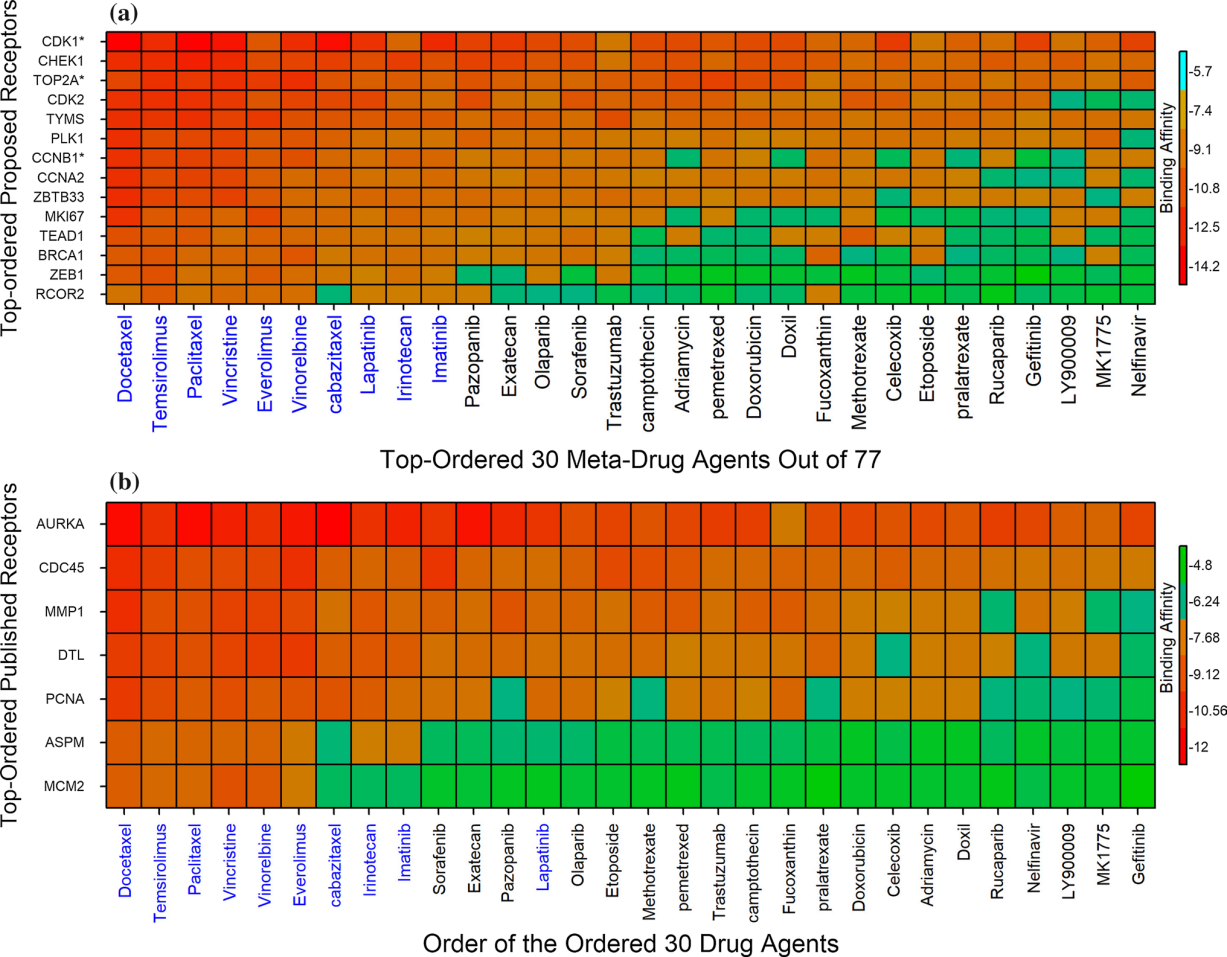
**a** Image of binding affinities based on the top-ordered 30 meta-drug agents out of 77 against the ordered 15 receptors, where red colors indicated the strong binding affinities, and the single star (*) indicated common receptors (published and proposed). **b** Image of binding affinity scores based on the ordered proposed 30 candidate-drugs in X-axis and Top-ordered 10 published proteins corresponding to CC in Y-axis

We considered 10 hHubGs and its regulatory 4 TFs (ZBTB33, TEAD1, ZEB1, RCOR2) as the m = 14 drug target receptors. We downloaded 3D structure of 14 receptors (BRCA1, CCNB1, CDK1, TOP2A, CCNA2, CDK2, CHEK1, MKI67, PLK1, TYMS, RCOR2, TEAD1, ZBTB33, ZEB1) from Protein Data Bank (PDB) [107] with PDB IDs 1n5o, 2b9r, 4y72, 1zxm, 1h26, 1b38, 1nvq, 1r21, 1q4o, 1hw3, 4czz, 6im5, 6df5, 2e19 respectively. The PubChem database [109] was used to retrieve the 3D structures of 77 drugs. To determine the binding scores for each pair of target proteins and meta-drug agents, molecular docking analysis was performed between m = 14 target proteins and n = 77 meta-drug agents. Then, we sorted the targets according to the row sums of the binding score matrix A = (Aij) and the drug agents according to the column sums in order to choose a small number of drug agents as the candidate drugs. The Fig. 5a showed the image of binding score matrix ${{\varvec{A}}}^{*}=\left({A}_{ij}^{*}\right)$ according to the sorted target proteins in Y-axis and n = 77 top ranked meta-drug agents in X-axis. We considered potential drug agents with a binding score of − 7.3 or less as better compounds against 14 targets. Therefore, we selected 10 top-ranked compounds (Docetaxel, Temsirolimus, Paclitaxel, Everolimus, Vincristine, Vinorelbine, cabazitaxel, Lapatinib, Irinotecan, Imatinib) as the possible candidate drug agents, possibly inhibiting the all proposed receptors for CC and these ten drugs are approved by U.S. Food and Drug Administration (FDA).

To validate our offered candidate drug agents by docking analysis with the already published targets related with CC infections, we picked up 54 papers on CC infections, those provided hub-genes and found only 10 hub-genes (ASPM, DTL, MMP1, TOP2A, PCNA, CCNB1, MCM2, AURKA, CDK1, CDC45) in which each of them was common within 4 articles out of 54 (see Table S1). We considered 10 targets corresponding to these 10 hub-genes to validate our selected drug agents against CC infections through docking analysis. From these 10 hub-genes, we observed that 3 genes (TOP2A, CDK1, CCNB1) were common with our selected hHubGs. So, the 3D structures of remain 6 hub-genes (DTL, MMP1, AURKA, PCNA, CDC45, MCM2) were downloaded from PDB with IDs 6qc0, 1cge, 1mq4, 1u76, 5dgo, 4uuz, respectively. The remain 3D structure of ASPM target was downloaded from UniProt [123] with sources ID of Q8IZT6. Then we performed docking analysis between top-ranked 30 drugs and published top-ranked 10 targets associated with CC infections. Their binding affinities (kcal/mol) were pictured in Fig. 5b. We accomplished that top-ranked 9 candidate drugs were common with our proposed top-ranked 10 candidate drugs. Finally we considered, the top-ranked 6 candidate drugs (Docetaxel, Temsirolimus, Paclitaxel, Vincristine, Everolimus, Vinorelbine) with binding affinities − 7.4 kcal/mol ≤ against the 7 published targets.

**Fig. 6:**
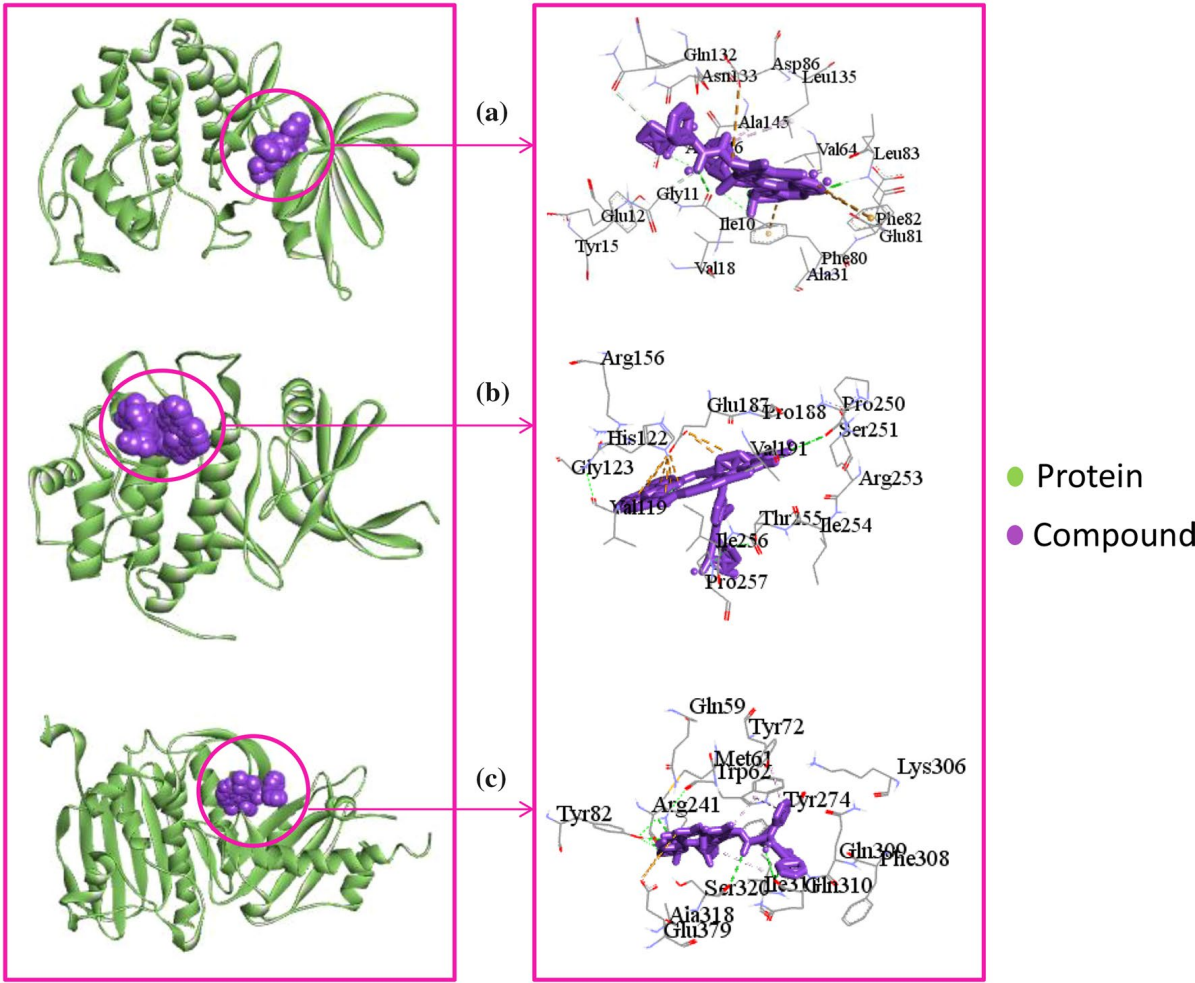
The top-ranked three complexes obtained from molecular docking study and its 2D chemical interactions. The figure is generated by using discovery studio visualizers. Complexes: **a** indicated CDK1_Docetaxel, **b** indicated CHEK1_Temsirolimus, and **c** indicated TOP2A_Paclitaxel

We considered top-ranked three docked complexes for protein-drug interaction profiling (see Table 2). As shown in Fig. 6a, CDK1_Docetaxel complex showed five hydrogen bonds with Leu83, Ile10, Gly11, Gln132, and Phe80 residues. Though the ligand formed major hydrophobic interactions with only two (Ala145, Leu135) residues, and three residues (Asp86, Phe80, Phe82) showed electrostatic interactions with the ligand. In Fig. 6b, CHEK1_Temsirolimus complex exposed two hydrogen bonds with Thr255, Pro250 residues, and the ligand formed electrostatic interactions with two (Glu187, Pro250) residues. In the case of the TOP2A_Paclitaxel complex, Paclitaxel showed six hydrogen bonds with Tyr82, Arg241, Arg241, Gln310, Ser320, and Gln310 residues. Paclitaxel also formed hydrophobic interactions with Ile311, Trp62, Tyr72 residues. Furthermore, Paclitaxel also formed electrostatic interactions with Glu379 residue (see Fig. 6c).

**Table 2:** Analysis of the interacting amino acids for the top three docked complex with top hit three compounds

Name of Complex	Interaction Amino Acids	Hydrogen Bonds	Hydrophobic Interactions	Electrostatic
CDK1_Docetaxel	Ile10, Gly11, Glu12, Tyr15, Val18, Ala31, Val64, Phe80, Glu81, Phe82, Leu83, Asp86, Gln132, Asn133, Leu135, Ala145, Asp146	Leu83, Ile10, Gly11, Gln132, Phe80	Ala145, Leu135	Asp86, Phe80, Phe82
CHEK1_Temsirolimus	Val119, His122, Gly123, Arg156, Glu187, Pro188, Val191, Pro250, Ser251, Arg253, Ile254, Thr255, Ile256, Pro257	Thr255, Pro250	-	Glu187, Pro250
TOP2A_Paclitaxel	Gln59, Met61, Trp62, Tyr72, Tyr82, Arg241, Tyr274, Lys306, Phe308, Gln309, Gln310, Ile311, Ala318, Ser320, Glu379	Tyr82, Arg241, Arg241, Gln310, Ser320, Gln310	Ile311, Trp62, Tyr72	Glu379

### Screening drugs with proposed hHubGs for CC treatment via DGIdb

**Fig. 7:**
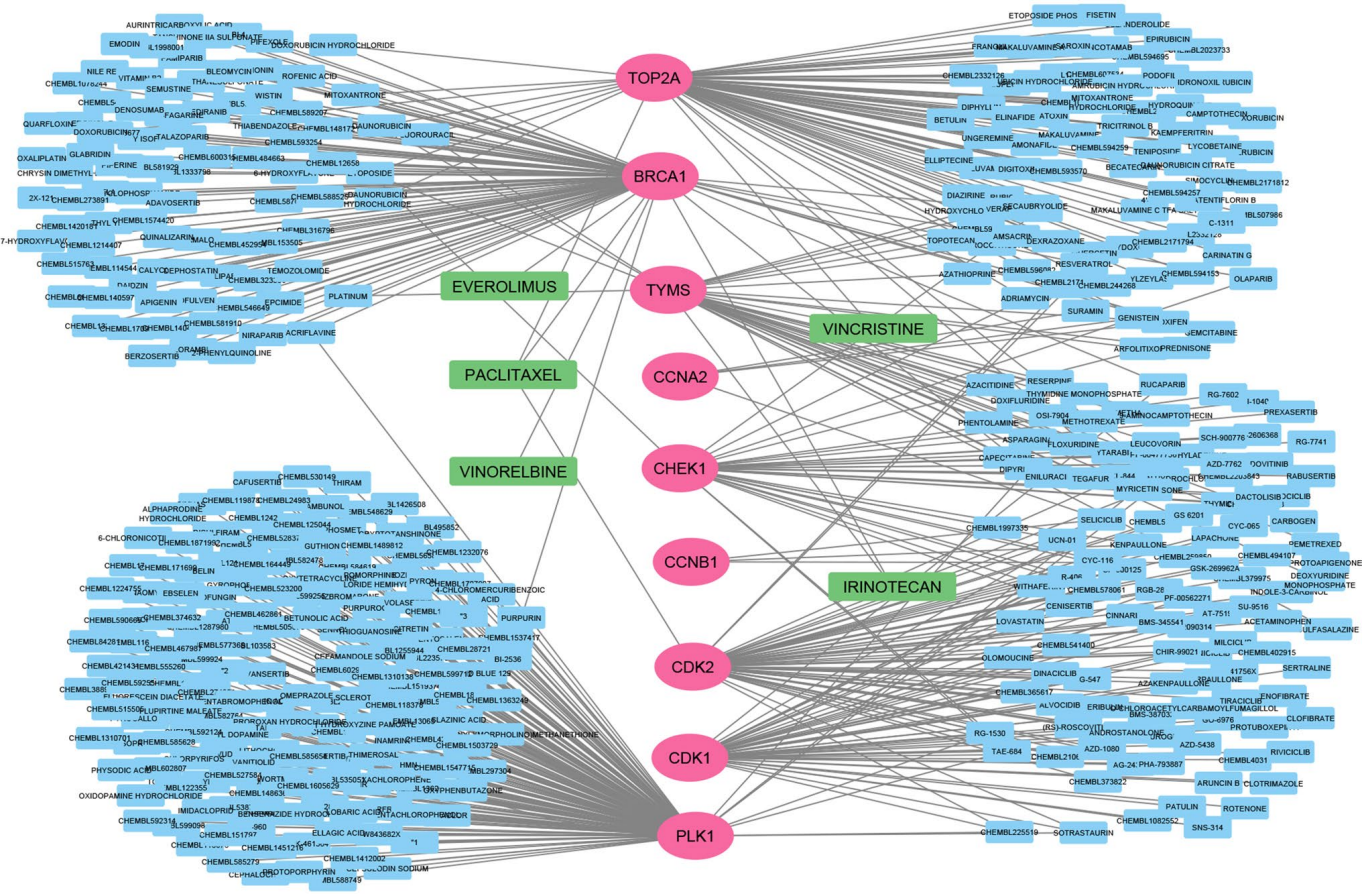
Gene-Drug interactions based on DGIdb and the network consists of 467 nodes and 539 edges. It contains proposed top-ranked 10 hHubGs (magenta color), drugs (deep sky blue) and the five drugs in light green (Paclitaxel, Vincristine, Everolimus, Vinorelbine, Irinotecan) indicated common drugs along with our proposed top-ranked 10 drugs

For the corss validation, Drug Gene Interaction Database (DGIdb) web resource [124] was used to retrieve drugs that interact with top-ranked 10 hHubGs. We found 9 out of the 10 genes have 456 interactions with drugs (see Fig. 7 and Supplementary Table S5). We found that, five drugs (Paclitaxel, Vincristine, Everolimus, Vinorelbine, Irinotecan) were common with our proposed top-ranked 10 drugs. BRCA1 has the highest number of inhibitory interactions among all genes with 5 drugs such as everolimus, vinorelbine, paclitaxel, and irinotecan. TOP2A can be inhibited by two drugs such as vincristine and paclitaxel. TYMS also can be inhibited by two drugs such as vincristine and irinotecan. CDK2 can be inhibited by paclitaxel.

### Molecular dynamic simulation

Docetaxel, Temsirolimus, and Paclitaxel were the top-ranked three potential drugs out of all those suggested. Thus, these top-ranked 3 drug-agents were considered to analyze the stability through molecular dynamic simulations.

**Fig. 8:**
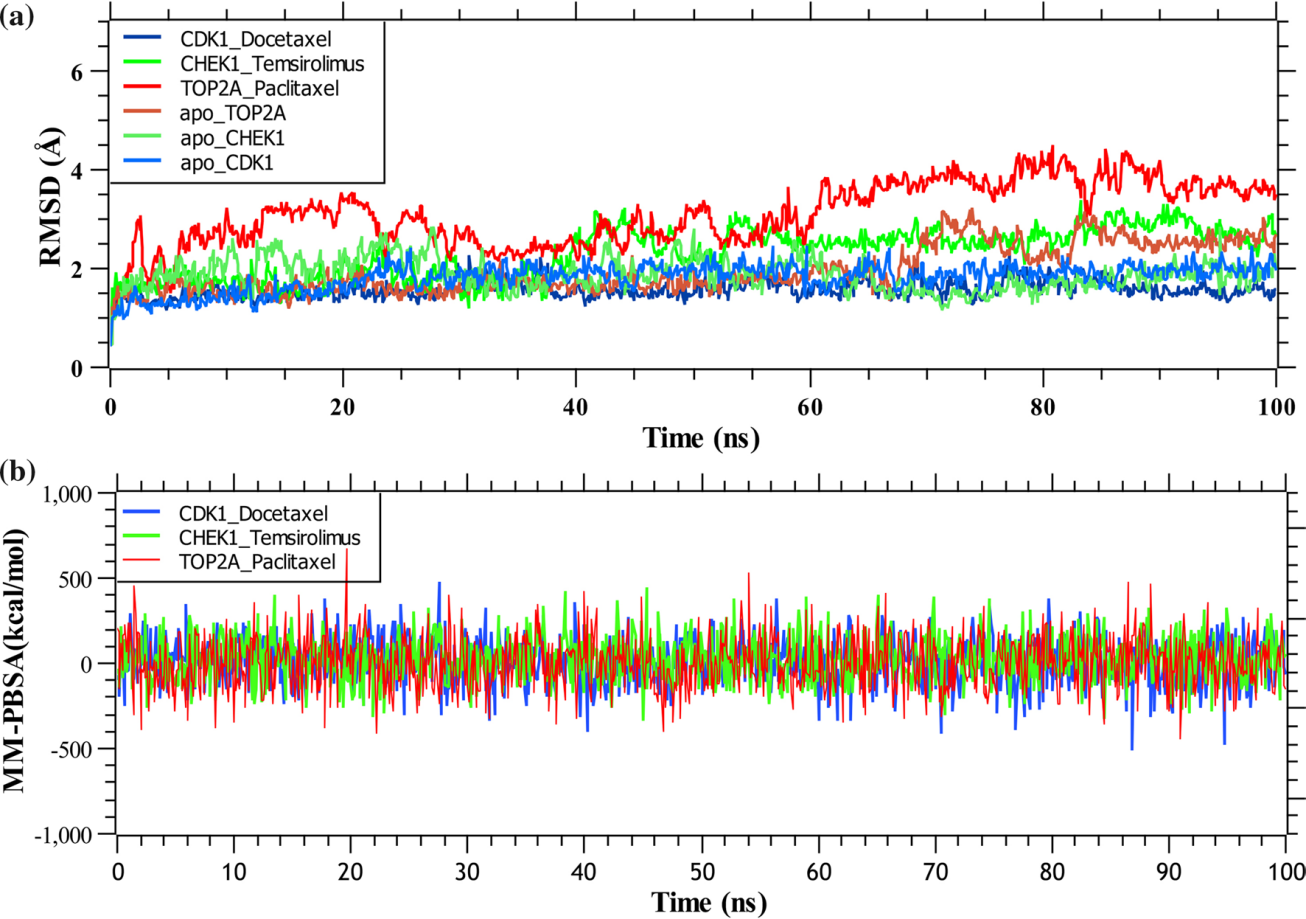
**a** Time evolution of root-mean-square deviations (RMSDs) of backbone atoms (C, Cα, and N) for protein for top-ranked three complexes. **b** Molecular mechanics Poisson-Boltzmann surface area (MM-PBSA) analysis was used to determine the binding free energy (kJ/mol) of each snapshot, which represents the change in binding stability of each complex across simulations; positive values indicate better binding. Complexes: Dark blue docetaxel-CDK1, green temsirolimus-CHEK1, red paclitaxel-TOP2A, light blue apo-CDK1, light green apo-CHEK1, light red apo-TOP2A

The RMSDs of three complexes and three apo form of proteins were determined from the simulation trajectories and plotted in Fig. 8a. From the figure, we observed that Paclitaxel-TOP2A obtained the highest RMSD values, while Docetaxel-CDK1 attained the lowest RMSDs in the 100 ns simulation. The figure demonstrates that docetaxel-CDK1 displayed a more stiff conformation compared to the other two proteins, reached equilibrium at 2 ns, and remained stable after that. On the other hand, the apo form of CDK1 also reached at equilibrium after 2 ns and maintained RMSD values up to 1.95 Å for 100 ns. The temsirolimus-CHEK1 complex exhibited slight fluctuations between about 40000 ps and 55000 ps and stabilized in the remaining simulations, and the apo form of CHEK1 also exhibited slight fluctuations between about 25000 ps and 50000 ps, after that it decreased to 1.4 Å and remained steady thereafter. On the contrary, the flexibility of paclitaxel-TOP2A complex increased dramatically, with RMSD values steadily increasing from 2 to 4 over time. The apo form of TOP2A reached at equilibrium after 3 ns and maintained RMSD up to 1.8 Å for 75 ns, after which the RMSD was seen to increase gradually. Here, we estimated the MM-PBSA binding energies for top-ranked 3 drugs with as mentioned previously. Figure 8b showed the binding energies for top-ranked 3 complexes (docetaxel-CDK1, temsirolimus-CHEK1, paclitaxel-TOP2A). The average binding energies of the docetaxel-CDK1, temsirolimus-CHEK1, paclitaxel-TOP2A complexes were 10.23 kJ/mol, 26.65 kJ/mol, and 7.56 kJ/mol, respectively.

## Discussion

CC is the most prevalent type of malignant tumor in women where its 5-year survival rate is only about 52%. Thus, for enhancing the survival rate and minimizing the mortality rate of CC patients it is required more studies to confirm the effective biomarkers and candidate drug agents [24]. For that, the target-based therapy for cervical cancer has become a hot spot in the medication system [125]. In recent years, multiple biomarkers for cervical cancer have been discovered (see Table S2). However, the complex and varied biomarkers of targeted therapy for cervical cancer also need to be properly uncovered and investigated [126]. In this study, we collected 255 hub genes/ study genes from 52 reputed published articles to find the independent meta-receptors which closely connected with CC disease. Among them, we detected 10 hHubGs (CDK1, CDK2, CHEK1, MKI67, TOP2A, BRCA1, PLK1, CCNA2, CCNB1, TYMS) as the proposed hub/key genes with emphasis on their roles, regulatory mechanisms, and therapeutic candidates. The box-plots based on the expression profile of hHubGs through the GEPIA web server showed that hHubGs are significantly differentially expressed genes (DEGs) between CC disease and control samnples. It should be noted here that the GEPIA web server was developed based on the RNA sequencing expression profiles of tumors and normal (9,736 and 8,587)samples comprising the TCGA and the GTEx projects.

According to the existing literature, CDK2 was a more promising therapeutic target for cervical cancer [37, 59]. CHEK1 hub gene produced a high score expression value in CC tissues compared with normal tissues and was considered as a significant upregulated expressed gene (P < 0.01) [20, 44]. Yuan et al. revealed that MKI67 showed high expression in CC [44]. TOP2A is considered a potential biomarker to improve the diagnosis of CC [12, 13, 20, 24, 32, 40, 42, 44, 55, 56]. CDK1 may play a significant role in regulating the genetic network associated with cervical cancer occurrence, development, and metastasis [20, 25, 32, 40–42, 45, 57, 65]. PLK1 played a role in the occurrence and progression of CSCC [45]. To activate the JAK/STAT pathway BRCA1 increased the sensitivity of cervical squamous cell carcinoma (CSCC) patients to cisplatin-based CCRT with upregulated expression of STAT1 [54]. CCNB1 also enacted a key role in the development of CC under some signaling pathways [12, 25, 32, 53, 65]. Y. Liu et al. reported that the progression of CC is activated by the expression of CCNA2 [41]. Some researchers discovered that TYMS expression levels were elevated in cervical cancer and were positively connected with cervical cancer prognosis [13, 24, 57].

We considered the top five mutual GO terms including BPs, MFs and CCs, and KEGG pathways to explore the pathogenetic processes of hHubGs, those were significantly associated with cervical cancer disease based on the hub-DEGs and hHubGs. Among them the GO terms, the top five common BPs (DNA replication, cell division, G1/S transition of mitotic cell cycle, mitotic nuclear division, regulation of signal transduction by p53 class mediator) showed the significant association with CC which was supported by the previous individual studies [12, 13, 127, 128]. The top five common MFs (protein binding, chromatin binding, ATP binding, protein kinase binding, protein heterodimerization activity) were significantly associated with CC disease and that were also supported by some previous studies[12, 13, 129–132]. Similarly, top four Cellular Components (nucleoplasm, cytosol, nucleus, spindle pole, and cytoplasm) were significantly associated with CC disease which was existed by the different individual literatures [133–135]. We also found the top five common KEGG pathways (Cell cycle, Pathways in cancer, HTLV-I infection, Hepatitis B, and p53 signaling pathway) that were significantly enriched for CC, and it is reported by some others [12, 42, 136].

We introduced four transcriptional (TFs) and three post-transcriptional (miRNAs) regulatory factors in results sections as well as the TFs proteins (TEAD1, ZBTB33, RCOR2, and ZEB1) were used as the drug target receptors for predicting the drug agents. In literature review, the previous study suggested that TEAD1 was an significant biomarker for CC [137]. ZBTB33 was able to prevent cervical cancer cell proliferation and EMT [138]. RCOR2 was established to be functional in cancer stem cells, where it positively regulates stemness gene expression [139]. Chen J. X. et al. suggested that the effect of hypoxia-induced ZEB1-driven cancer cells induced macrophage infiltration into hypoxic area through the CCR2–NF-κB pathway showed poor connection of prognosis in CC [140].

**Fig. 9:**
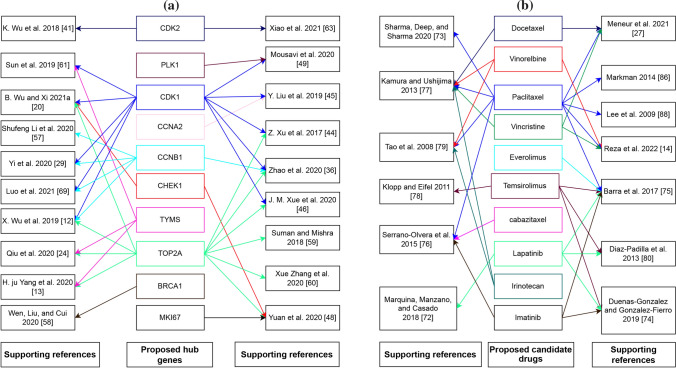
**a** Proposed CC-causing hub genes (hHubGs) with supporting references, where a specific color indicates the references for a specific hub-gene. **b** Proposed candidate drugs (FDA approaved) with supporting references, where a specific color indicates the references for a specific candidate drug

We considered top-ranked identified proteins and their regulation 4 important TF proteins as drug target proteins and conducted molecular docking analysis with 77 meta-drug agents to find viable candidate drug agents for the medication of CC (see Table S1). Then, we choose top-ranked ten drugs (Docetaxel, Temsirolimus, Paclitaxel, Vincristine, Everolimus, Vinorelbine, cabazitaxel, Lapatinib, Irinotecan, Imatinib) as the potential drugs for CC infections based on their binding affinity scores (kCal/mol) compared with all the target receptors (see Fig. 5a). On the other hand, we also confirmed that our proposed hHubGs suggested by others. Moreover, the hHubGs matched by previous articles in favour of 10 studies for TOP2A and CDK1, 5 studies for CCNB1, 3 studies for TYMS, 2 studies for CDK2 and CHEK1, and only single study for PLK1, CCNA2, BRCA1and MKI67 genes (see Fig. 9a). The suggested drugs of our study also reported by the other researchers such as Docetaxel [70, 91], Temsirolimus [72, 74, 88, 89], Paclitaxel [14, 70, 73, 80, 82, 87, 89, 91], Vincristine [14, 70, 91], Everolimus [89], Vinorelbine [14, 73, 91], cabazitaxel [90], Lapatinib [74, 86, 88, 89], Irinotecan [73, 91], Imatinib[88–90] for the treatment of CC infections (see Fig. 9b). On the other hand, we used DGIdb online database to screen the drugs that interact with our proposed 10 hHubGs for cross validation and found that five drugs (Paclitaxel, Vincristine, Everolimus, Vinorelbine, Irinotecan) were common with our proposed top-ranked 10 drugs. Then, we also validated top-ranked 30 candidate-drugs against the top-ranked 10 published receptors (ASPM, DTL, MMP1, AURKA, PCNA, CCNB1, CDC45, MCM2, TOP2A, CDK1) associated with CC infections by molecular docking analysis and found their strongly significant binding affinity scores with top-ranked 6 candidate-drugs (see Fig. 5b). Finally, we investigated the stability of top-ranked three drugs (Docetaxel, Temsirolimus, Paclitaxel) by using 100 ns MD-based MM-PBSA simulations for three top-ranked proposed receptors (CDK1, CHEK1, TOP2A), and observed their stable performance according to the laws of physics [141, 142]. Therefore, the proposed candidate drugs might be played a vital role in the treatment of CC infections.

## Conclusions

The current work used a variety of well-known bioinformatics tools to discover hub of the HubGs (hHubGs) highlighting their regulatory factors and dysregulated molecular functions and pathways pathways that are responsible for CC development. At first, we collected 255 HubGs/studied-genes that were published by the individual studies.. Among them, we selected 10 HubGs (CDK1, CDK2, CHEK1, MKI67, TOP2A, BRCA1, PLK1, CCNA2, CCNB1, TYMS) as the hHubGs by the PPI network analysis and validated their differential expression patterns between CC and normal samples through the GPEA database. The gene ontology (GO) and KEGG pathway enrichment analysis of HubGs revealed some crucial CC-causing BPs (DNA replication, cell division), MFs (protein binding, chromatin binding) and CCs (nucleoplasm, cytosol) by involving hHubGs. The gene regulatory network (GRN) analysis exposed four TFs proteins (TEAD1, ZBTB33, RCOR2, and ZEB1) and three miRNAs (hsa-miR-548d-5p, hsa-miR-146a and hsa-miR-559) as the key transcriptional and post-transcriptional regulators of hHubGs. Then, we identified hHubGs-guided top-ranked FDA-approved 10 candidate drugs (Docetaxel, Temsirolimus, Paclitaxel, Vincristine, Everolimus, Vinorelbine, cabazitaxel, Lapatinib, Irinotecan, Imatinib) and validated them against the state-of-the-arts independent receptors by molecular docking analysis. Finally, we offered possible candidate drug agents, such as Docetaxel, Temsirolimus, Paclitaxel, and examined their stability performance by using 100 ns MD-based MM-PBSA simulations for the top-ranked three proposed proteins (CDK1, CHEK1, TOP2A), and also detected their stable performance. Hence, the selected genetic biomarkers and candidate repurposing drugs derived from this study has merit for CC disease diagnosis and therapies.

## Supplementary Information

**Additional file 1: Table S1**. Different hub/studied genes list for CC infection published by different papers in different international reputed journals. **Table S2**. 77 meta-drug agents for the treatment against CC. **Table S4**. The top 20 significantly (p-value<0.001) enriched GO functions and KEGG pathways by cDEGs involving KGs with CC diseases. **Table S5.** Gene-Drug interactions analysis based on our proposed hHubGs using DGIdb database.

**Additional file 2: Table S3**. The MCODE raw file was selected from the PPI network through Cytoscape software, where the degree cutoff was used 80.

## Data Availability

All data analyzed during this study are included in this article and its additional files.

## Abbreviations

CC: Cervical cancer
CSCC: Cervical squamous cell carcinoma
HPV: Human Papillomavirus
PPI: Protein–Protein Interaction
ENCODE: Encyclopedia Of DNA Elements
MCODE: Molecular Complex Detection
DEGs: Differentially Expressed Genes
cDEGs: Common Differentially Expressed Genes
cHubGs: Common Hub Genes
cHubPs: Common Hub Proteins
hHubGs: Hub of the hub-genes
KPs: Key proteins
GO: Gene ontology
BPs: Biological processes
MFs: Molecular function
CCs: Cellular components
KEGG: Kyoto Encyclopedia of Genes and Genomes
TFs: Transcription factors
miRNAs: Micro-RNAs
MD: Molecular dynamic
MM-PBSA: Molecular Mechanics Poisson–Boltzmann Surface Area
RMSD: Root mean square deviation
3D: Three-Dimensional
PDB: Protein data bank
FDA: U.S. Food and Drug Administration
YASARA: Yet Another Scientific Artificial Reality Application
