# Supplementary material for: Metadata analysis to explore hub of the hub-genes highlighting their functions, pathways and regulators for cervical cancer diagnosis and therapies
Source: Discov Oncol. 2022 Aug 22;13:79. doi: 10.1007/s12672-022-00546-6 (PMC9395557; doi:10.1007/s12672-022-00546-6)
Supplement: Supplementary file 1 — Additional file 1: Table S1. Different hub/studied genes list for CC infection published by different papers in different international reputed journals. Table S2. 77 meta-drug agents for the treatment against CC. Table S4. The top 20 significantly (p-value<0.001) enriched GO functions and KEGG pathways by cDEGs involving KGs with CC diseases. Table S5. Gene-Drug interactions analysis based on our proposed hHubGs using DGIdb database. [file 12672_2022_546_MOESM1_ESM.docx]

**Table S1:** Different hub/studied genes list for CC infection published by different papers in different international reputed journals.

| **Publications** | **Collected hub /study genes** | **Common hub/study genes with at least 4 articles** |
| --- | --- | --- |
| (Jing Feng 2018) [1] | PCNA, CDK4, Cyclin D1, p21, E-cadherin, β-catenin, Vimentin, U6, GAPDH, ZBTB33 |  |
| (Venkataramnan and Binti Zainol Izam Khan 2020)[2] | AURKA, UBE2C, TPX2, MCM5, GINS2 | ASPM, DTL, MMP1, AURKA, PCNA, CCNB1, CDC45, MCM2, TOP2A, CDK1 |
| (Y. Mei et al. 2020)[3] | APOBEC3B, DSG2, CXCL8, ABCA8, PLAGL1 |  |
| (Jinhui Liu et al. 2019)[4] | EPHX2, RMI2 |  |
| (Qiu et al. 2020)[5] | RRM2, CDC45, GINS2, HELLS, KNTC1, MCM2, MYBL2, PCNA, RAD54 L, RFC4, RFC5, TK1, TOP2A, TYMS |  |
| (Meneur et al. 2021)[6] | FASN, HK2, ACACB, PIF1, COX7A1, CKMT2, CPT1B, PDK4, DNA2, and COX4I2 |  |
| (X. Wu et al. 2019)[7] | TSPO, CCND1, FOS, CDK1, TOP2A, CCNB1, PCNA, BIRC5, MAD2L1 |  |
| (J. Mei et al. 2020)[8] | RIPOR2, CXCL8 |  |
| (Yi et al. 2020)[9] | CDK1, CCNB1, ITGB1, FN1, MMP9, STAT1 |  |
| (Deng, Zhu, and Huang 2016)[10] | GPS1, CCDC65, SLC9A7, VARS, SNX3, ENPP4, RGS7, LOC125943, LOC143903, LOC166602, BICC1, LOC253956, LOC220362, LOC206117, ADA, NAV1, LEF1 |  |
| (H. ju Yang et al. 2020)[11] | TYMS, MCM2, HELLS, TOP2A, CXCL8 |  |
| (J. Wang et al. 2020)[12] | DSG2, MMP1, SPP1, MCM2 |  |
| (Jinhui Liu, Yang, et al. 2020)[13] | EZH2, FLT1, GAPDH |  |
| (Ouyang et al. 2020)[14] | SNRPA, CCDC12 |  |
| (H. Chen et al. 2020)[15] | KNTC1 |  |
| (H. Xue et al. 2021)[16] | CDC45, GINS2, MCM2, PCNA |  |
| (Zhao et al. 2020)[17] | CDC6, CDK1, CDC45, BUB1, TOP2A, MCM4, CCNB2, CCNB1 |  |
| (Ma et al. 2020)[18] | ESR1, EPB41L3, EDNRB, ID4, PLAC8 |  |
| (Mallik et al. 2020)[19] | PAIP2, GRWD1, VPS4B, CRADD, LLPH, MRPL35, FAM177A1, STAT4, ASPSCR1, FABP7 |  |
| (Jinhui Liu, Li, et al. 2020)[20] | ACKR1 |  |
| (Tu et al. 2021)[21] | RFC5, POLE3, RAD51, RMI1, PALB2, HDAC1, MCM4, ESR1, FOS, E2F1 |  |
| (K. Wu et al. 2018)[22] | PCNA, CDK2, VEGFA, PIK3CA |  |
| (Jinhui Liu, Wu, et al. 2020)[23] | CD1C, CD6 |  |
| (Jiamei Liu, Liu, and Yang 2021)[24] | NDC80, TIPIN, MCM3, MCM6, POLA1, PRC1 |  |
| (Z. Xu et al. 2017)[25] | CDK1, TOP2A, MCM2, AURKA, KIF20A, ESR1 |  |
| (Y. Liu et al. 2019)[26] | CCNA2, CDK1, CCND1, FGF2, IGF1, BCL2, VEGFA |  |
| (J. M. Xue et al. 2020)[27] | TOP2A, ECT2, RRM2, ANLN, NEK2, ASPM, BUB1B, CDK1, DTL, PRC1 |  |
| (M. Wang et al. 2018)[28] | MCM2, MMP2, COL1A1, JUN |  |
| (Yuan et al. 2020)[29] | TOP2A, AURKA, CHEK1, KIF11, MCM4, MKI67, DTL, FOXM1, SMC4, FBXO5, |  |
| (Mousavi et al. 2020)[30] | CDK1, PLK1 |  |
| (Xi Zhang and Wang 2019)[31] | SMYD2, EGLN1, TNFRSF10D, FUT11, SYTL3, MMP8, EREG |  |
| (He et al. 2021)[32] | CDC45 |  |
| (Q. Chen et al. 2018)[33] | TP53, MMP1, NOTCH1, SMAD4, NFKB1 |  |
| (Shiyan Li et al. 2021)[34] | SLC25A5, ENO1, ANLN, RIBC2, PTTG1, MCM5 |  |
| (Fu, Wu, and Xue 2018)[35] | ARFGAP3 |  |
| (B. Wu and Xi 2021b)[36] | CDC45, ORC1, RPA1, CDT1, TARDBP, RBMX, SRSF3, SRSF1, RFC5, RFC2, MSH6, DTL, MSH2 |  |
| (Meng et al. 2020)[37] | DSG2, ITM2A, CENPM, RIBC2, MEIS2 |  |
| (Ding et al. 2020)[38] | PTK2, NRP1, PRKAA1, HMGCS1 |  |
| (Shufeng Li et al. 2020)[39] | CCNB1 |  |
| (B. Wu and Xi 2021a)[40] | NUSAP1, TOP2A, KIF2C, NDC80, ASPM, KIF20A, CDK1, KIF11, BIRC5, MCM2, CHEK1 |  |
| (Wen, Liu, and Cui 2020)[41] | BRCA1, CDCA8, ASPM, CDC45, RAD51, HMMR, CENPF, EXO1, DTL, ZWINT |  |
| (Suman and Mishra 2018)[42] | TOP2A, BIRC5, AURKA, CCNB2 |  |
| (Xue Zhang et al. 2020)[43] | ASPM, CDC45, CDC7, CENPO, PRIM2, RFC4, EXO1, TOP2A, CENPQ, RAD54L, ZWILCH, NCAPH, KIAA0101, TOPBP1 |  |
| (Sun et al. 2019)[44] | TYMS, SASH1, CDK1, AURKA |  |
| (Oany et al. 2021)[45] | PTPRC, ITGAM, IL10, TYROBP, ITGB2, CCR5, ITGAX, CSF1R, LILRB2, CXCR4, STAT3, and CYBB |  |
| (Xiao et al. 2021)[46] | RFC4, ATAD2, TRIP13, NUF2, FOXM1, ECT2, KIF14, CDK2, KNTC1, DNA2. |  |
| (Yu et al. 2018)[47] | IGF2R, DNAJC6, FZD4, CBL, CLTC, ARPC3, VAMP2, ITGB3, VAMP4, ITGB1, MAPK8, KIF2A, CLASP1, SMC3, STAG2, STAG1, H2AFZ, RBBP4, UBE2D3 |  |
| (Xiaoyu Zhang et al. 2019)[48] | JUN, DCN, THBS1, HLA-DRA, EDN1, TIMP3, TGFB2, OAS3, OASL, OAS1, PDGFB, MMP1, EGR1, SDC4, SERPINE1 |  |
| (F. Xu, Shen, and Xu 2021)[49] | CCR7, CD3D, CD3E, ITGB2, FAM133A, TP53 |  |
| (Jiang et al. 2021)[50] | APOD, CXCL8, MMP1, MMP3, PLOD2, PTGDS, SNX10, SPP1 |  |
| (C. Yang, Xu, and Jin 2016)[51] | PBXl, LAMC2 |  |
| (Luo et al. 2021)[52] | CDK1, BUB1, KIF11, NDC80, BUB1B, CCNB2, PCNA, CCNB1, MAD2L1 and CDCA8, |  |
| (Tong et al. 2021)[53] | RAD23A, PGD, ITPA, IDH1, THBS1 |  |
| (Z. Zhang et al. 2021)[54] | SELL, CX3CR1, FCGR2B, CD38, IL15, HLA-DRA, CCL8, CD79A, HLA-DMA, HLA-DPA1 |  |

**Table S2:** 77 meta-drug agents for the treatment against CC.

| **Candidate drug agents Name** | **Reference** |
| --- | --- |
| 5_Fluorouracil, Adriamycin, Bleomycin, Bleomycin_Sulfate, Brivanib,Celecoxib, Cetuximab, Chlorambucil, Ciclopirox, Curcumin, Cyclophosphamide, Decitabine, Docetaxel, Doxil, Doxorubicin, Eribulin_Mesylate, Erlotinib, toposide, Everolimus, Exatecan, Fluorouracil, Fucoxanthin, Gefitinib, Geldanamycin, Gemcitabine, Hycamtin, Ifosfamide, Imatinib, Irinotecan, LY900009, Lapatinib, MK1775, Melphalan, Mesna, Methotrexate, Mitolactol, Mitomycin_C, Nelfinavir, Olaparib, Paclitaxel, Pazopanib, Piperlongumine, Rucaparib, Sizofiran, Sorafenib, Sunitinib, TNP_470, Temsirolimus, Thioridazine, Tirapazamine, Topotecan, Topotecan_Hydrochloride, Trastuzumab, Triapine, Veliparib, Vincristine, Vinorelbine, Vorinostat, amifostine, apigenin, belotecan, bendamustine, cabazitaxel, camptothecin, carboplatin, chrysin, cisplatin, gimeracil, hydralazine, hydroxyurea, pemetrexed, pralatrexate, resveratrol, tegafur, thioguanosine, thrombospondin_1, trichostatin_A, valproate, vedotin | [3,4,61–70,5,71–74,6,55–60] |

**Table S4:** The top 20 significantly (p-value<0.001) enriched GO functions and KEGG pathways by cDEGs involving KGs with CC diseases.

| **Biological Process** | | | | | |
| --- | --- | --- | --- | --- | --- |
| **Term** | **Count** | **PValue** | **cDEGs** | **FDR** | |
| GO:0006260~DNA replication | 18 | 1.58E-16 | RFC5, GINS2, FEN1, RFC4, RRM2, MCM7, CDC7, BRCA1, CHAF1A, MCM3, TIMELESS, CDK1, MCM4, MCM5, TOPBP1, MCM6, DTL, MCM2 | 1.43E-13 | |
| GO:0051301~cell division | 22 | 7.94E-15 | SPAG5, UBE2C, NCAPG2, KIF14, NCAPG, CDC7, SMC4, ZWINT, AURKA, CDC20, TPX2, CENPF, CCNB1, KIF18B, CKS2, TIMELESS, CDK1, BIRC5, KNTC1, MCM5, NEK2, KIF2C | 3.57E-12 | |
| GO:0000082~G1/S transition of mitotic cell cycle | 12 | 5.61E-11 | DHFR, RRM2, MCM7, CDKN2A, CDK1, MCM3, MCM4, CDC7, MCM5, MCM6, TYMS, MCM2 | 1.68E-08 | |
| GO:0006270~DNA replication initiation | 8 | 1.18E-09 | MCM7, MCM3, MCM4, CDC7, MCM5, TOPBP1, MCM6, MCM2 | 2.65E-07 | |
| GO:0007067~mitotic nuclear division | 13 | 7.29E-08 | NCAPG2, AURKA, CDC20, ASPM, TPX2, CENPF, TIMELESS, CDK1, BIRC5, KNTC1, NEK2, KIF2C, CENPN | 1.31E-05 | |
| GO:0006268~DNA unwinding involved in DNA replication | 5 | 3.42E-07 | TOP2A, MCM7, MCM4, MCM6, MCM2 | 4.72E-05 | |
| GO:0006281~DNA repair | 12 | 3.67E-07 | RFC5, RAD51AP1, FANCI, POLQ, FEN1, CHAF1A, CDK1, RAD54L, BRCA1, TOPBP1, FOXM1, FANCG | 4.72E-05 | |
| GO:0000086~G2/M transition of mitotic cell cycle | 9 | 3.04E-06 | TPX2, CCNB1, MELK, CDK1, BIRC5, NEK2, HMMR, FOXM1, AURKA | 3.04E-04 | |
| GO:0007059~chromosome segregation | 7 | 5.14E-06 | TOP2A, CENPF, SPAG5, HJURP, CENPN, NEK2, BRCA1 | 4.21E-04 | |
| GO:0008283~cell proliferation | 12 | 2.58E-05 | TPX2, AR, CENPF, DACH1, MELK, MCM7, CDK1, CKS2, EMP1, KIF2C, TYMS, DLGAP5 | 0.001938 | |
| GO:0007051~spindle organization | 4 | 1.40E-04 | ASPM, SPAG5, TTK, AURKA | 0.009699 | |
| GO:0048146~positive regulation of fibroblast proliferation | 5 | 4.14E-04 | CCNB1, PDGFD, ESR1, EREG, AQP1 | 0.021432 | |
| GO:0006974~cellular response to DNA damage stimulus | 8 | 4.15E-04 | TOP2A, POLQ, MCM7, TIMELESS, BRCA1, TOPBP1, DTL, FANCG | 0.021432 | |
| GO:0051439~regulation of ubiquitin-protein ligase activity involved in mitotic cell cycle | 4 | 4.29E-04 | CDC20, CCNB1, UBE2C, CDK1 | 0.021432 | |
| GO:0000083~regulation of transcription involved in G1/S transition of mitotic cell cycle | 4 | 4.29E-04 | DHFR, RRM2, CDK1, TYMS | 0.021432 | |
| GO:0070301~cellular response to hydrogen peroxide | 5 | 5.09E-04 | PDGFD, CDK1, ECT2, EZH2, AQP1 | 0.024028 | |
| GO:0007049~cell cycle | 8 | 5.34E-04 | CDC20, CHAF1A, GMNN, HJURP, BRCA1, FOXM1, AURKA, MCM2 | 0.024028 | |
| GO:0045840~positive regulation of mitotic nuclear division | 4 | 6.20E-04 | EDN3, NUSAP1, EREG, AURKA | 0.026591 | |
| GO:1901796~regulation of signal transduction by p53 class mediator | 6 | 0.001283 | RFC5, TPX2, RFC4, BRCA1, TOPBP1, AURKA | 0.05019 | |
| GO:0000724~double-strand break repair via homologous recombination | 5 | 0.001363 | RAD51AP1, POLQ, FEN1, RAD54L, BRCA1 | 0.051125 | |
| **Molecular Function** | | | | | |
| GO:0005524~ATP binding | 30 | 3.80E-08 | TOP2A, MCM7, GMPS, KIF14, TTK, ATP2C1, SMC4, AURKA, RAD54L, NEK2, RFC5, POLQ, RFC4, UBE2C, ATAD2, CDC7, ABCA8, TPX2, KIF18B, MELK, KIF4A, MCM3, CDK1, MCM4, MCM5, KIF2C, KIF20A, MCM6, ATP13A3, MCM2 | 9.69E-06 | |
| GO:0005515~protein binding | 83 | 2.65E-07 | TOP2A, SPON1, FEN1, MCM7, HSPB8, NCAPG2, GMNN, HJURP, KIF14, BRCA1, FOXM1, SMC4, AQP1, CDC20, CHAF1A, DACH1, NUSAP1, KNTC1, NEK2, WHSC1, TOPBP1, GTSE1, DLGAP5, RFC5, WDHD1, RFC4, KRT4, IGFBP5, KRT2, KRT1, EMP1, EREG, TGFBR3, AR, MELK, CKS2, MCM3, TIMELESS, MAL, BIRC5, MCM4, MCM5, KIF2C, KIF20A, MCM6, DTL, MCM2, NCAPG, TTK, ALOX12, HMMR, AURKA, RAD51AP1, SYNGR1, CCNB1, RAD54L, APOD, SLIT2, ECT2, FANCI, GINS2, POLQ, RRM2, JUP, SPAG5, CDKN2A, UBE2C, CDC7, SORBS1, ESR1, SHCBP1, FANCG, HOPX, ZWINT, TPX2, CENPF, KIF18B, KRT17, CENPI, PRC1, KIF4A, CDK1, EZH2 | 3.20E-05 | |
| GO:0003678~DNA helicase activity | 6 | 3.77E-07 | MCM7, MCM3, MCM4, MCM5, MCM6, MCM2 | 3.20E-05 | |
| GO:0003682~chromatin binding | 12 | 4.13E-05 | TOP2A, POLQ, AR, CENPF, CHAF1A, ATAD2, CDK1, CKS2, MCM5, WHSC1, ESR1, EZH2 | 0.002632 | |
| GO:0003677~DNA binding | 25 | 1.20E-04 | TOP2A, FEN1, MCM7, HJURP, BRCA1, FOXM1, DACH1, NUSAP1, RAD54L, TOPBP1, RFC5, FANCI, WDHD1, POLQ, RFC4, CDKN2A, ESR1, HOPX, AR, KIF4A, MCM3, MCM4, MCM6, MCM2, EZH2 | 0.006144 | |
| GO:0019901~protein kinase binding | 10 | 6.90E-04 | TPX2, CCNB1, JUP, CDKN2A, PRC1, KIF14, CKS2, KIF20A, FOXM1, AURKA | 0.029308 | |
| GO:0019899~enzyme binding | 9 | 0.001342 | RFC5, TOP2A, CDC20, AR, RFC4, BIRC5, BRCA1, ESR1, MCM2 | 0.048903 | |
| GO:0003777~microtubule motor activity | 5 | 0.001725 | KIF18B, KIF4A, KIF14, KIF2C, KIF20A | 0.050003 | |
| GO:0043142~single-stranded DNA-dependent ATPase activity | 3 | 0.001765 | RFC5, POLQ, RFC4 | 0.050003 | |
| GO:0008017~microtubule binding | 7 | 0.002198 | KIF18B, PRC1, KIF4A, NUSAP1, KIF14, BIRC5, KIF20A | 0.056039 | |
| GO:0008574~ATP-dependent microtubule motor activity, plus-end-directed | 3 | 0.00518 | KIF18B, KIF4A, KIF14 | 0.120084 | |
| GO:0016887~ATPase activity | 6 | 0.006431 | ATAD2, KIF14, KIF2C, KIF20A, ATP13A3, ABCA8 | 0.136663 | |
| GO:0042803~protein homodimerization activity | 12 | 0.007021 | TOP2A, GYS2, CENPF, UPK1A, JUP, HPGD, TIMELESS, BIRC5, TTK, SLIT2, TYMS, ECT2 | 0.137719 | |
| GO:0003684~damaged DNA binding | 4 | 0.007656 | POLQ, FEN1, BRCA1, FANCG | 0.139456 | |
| GO:0004003~ATP-dependent DNA helicase activity | 3 | 0.017752 | MCM7, MCM4, MCM6 | 0.301781 | |
| GO:0003697~single-stranded DNA binding | 4 | 0.021808 | RAD51AP1, MCM7, MCM4, MCM6 | 0.347565 | |
| GO:0035173~histone kinase activity | 2 | 0.025349 | CCNB1, CDK1 | 0.380229 | |
| GO:0015631~tubulin binding | 3 | 0.02957 | KIF14, BIRC5, BRCA1 | 0.41891 | |
| GO:0086083~cell adhesive protein binding involved in bundle of His cell-Purkinje myocyte communication | 2 | 0.037783 | JUP, DSG2 | 0.507089 | |
| GO:0042393~histone binding | 4 | 0.043499 | ATAD2, HJURP, CKS2, MCM2 | 0.533573 | |
| **Cellular Component** | | | | | |
| GO:0005654~nucleoplasm | 56 | 4.91E-17 | TOP2A, FEN1, MCM7, HSPB8, NCAPG2, GMNN, HJURP, BRCA1, FOXM1, SMC4, CDC20, WHSC1, TOPBP1, GTSE1, RFC5, WDHD1, RFC4, PEG3, AR, MCM3, TIMELESS, BIRC5, MCM4, MCM5, KIF20A, MCM6, DTL, MCM2, HPGD, TYMS, AURKA, RAD51AP1, CCNB1, RAD54L, FANCI, GINS1, GINS2, POLQ, RRM2, SPAG5, CDKN2A, UBE2C, ATAD2, CDC7, SORBS1, ESR1, FANCG, DHFR, TPX2, CENPF, CENPI, PRC1, KIF4A, CDK1, CENPN, EZH2 | 7.55E-15 | |
| GO:0030496~midbody | 13 | 2.54E-11 | SPAG5, KIF14, SHCBP1, AURKA, ASPM, CENPF, PRC1, KIF4A, CDK1, BIRC5, NEK2, KIF20A, ECT2 | 1.95E-09 | |
| GO:0042555~MCM complex | 6 | 1.04E-09 | MCM7, MCM3, MCM4, MCM5, MCM6, MCM2 | 5.32E-08 | |
| GO:0005634~nucleus | 65 | 2.18E-09 | TOP2A, FEN1, MCM7, HSPB8, NCAPG2, GMNN, FAM107A, HJURP, KIF14, BRCA1, FOXM1, SMC4, AQP1, CDC20, CHAF1A, DACH1, NUSAP1, KNTC1, NEK2, WHSC1, TOPBP1, DLGAP5, KRT4, PEG3, KRT2, KRT1, ASPM, AR, MELK, MCM3, TIMELESS, BIRC5, MCM4, MCM5, KIF2C, MCM6, DTL, MCM2, NCAPG, TYMS, AURKA, RAD51AP1, CCNB1, NDN, RAD54L, ECT2, GINS1, GINS2, RRM2, JUP, SPAG5, CDKN2A, ATAD2, CDC7, SORBS1, ESR1, HOPX, ZWINT, TPX2, CENPF, KIF18B, CENPI, PRC1, CDK1, EZH2 | 8.38E-08 | |
| GO:0005819~spindle | 10 | 6.21E-08 | CDC20, TPX2, CENPF, PRC1, NUSAP1, BIRC5, TTK, KIF20A, SHCBP1, AURKA | 1.91E-06 | |
| GO:0005876~spindle microtubule | 7 | 2.90E-07 | SPAG5, PRC1, KIF4A, NUSAP1, CDK1, BIRC5, AURKA | 6.74E-06 | |
| GO:0005737~cytoplasm | 59 | 3.07E-07 | TOP2A, MCM7, HSPB8, GMNN, HJURP, BRCA1, FOXM1, SMC4, AQP1, GYS2, CDC20, ENDOU, DACH1, NUSAP1, KNTC1, NEK2, WHSC1, TOPBP1, DLGAP5, WDHD1, KRT2, TGFBR3, ASPM, AR, BIRC5, DTL, MCM2, HPGD, GMPS, NCAPG, TTK, ALOX12, TYMS, CCNB1, SLIT2, ECT2, FANCI, GINS1, RRM2, JUP, SPAG5, CDKN2A, UBE2C, CDC7, SORBS1, ESR1, SHCBP1, FANCG, HOPX, ZWINT, CRNN, CENPF, KIF18B, KRT17, CENPI, PRC1, KIF4A, CDK1, EZH2 | 6.74E-06 | |
| GO:0000922~spindle pole | 9 | 3.84E-07 | CDC20, TPX2, CENPF, CCNB1, SPAG5, PRC1, KNTC1, NEK2, TOPBP1 | 7.40E-06 | |
| GO:0000777~condensed chromosome kinetochore | 8 | 1.13E-06 | SPAG5, HJURP, BIRC5, KNTC1, KIF2C, CENPN, NEK2, ZWINT | 1.94E-05 | |
| GO:0005694~chromosome | 8 | 4.03E-06 | POLQ, KIF4A, NUSAP1, BRCA1, TOPBP1, WHSC1, SMC4, DTL | 6.21E-05 | |
| GO:0000776~kinetochore | 7 | 1.11E-05 | CENPF, SPAG5, CENPI, TTK, KIF2C, NEK2, ZWINT | 1.55E-04 | |
| GO:0000784~nuclear chromosome, telomeric region | 8 | 1.65E-05 | FEN1, MCM7, CDK1, MCM3, MCM4, MCM5, MCM6, MCM2 | 2.12E-04 | |
| GO:0005813~centrosome | 12 | 6.86E-05 | CDC20, CENPF, CCNB1, CDK1, MCM3, NDN, NCAPG, NEK2, TOPBP1, SORBS1, DTL, AURKA | 8.12E-04 | |
| GO:0005874~microtubule | 10 | 1.38E-04 | TPX2, KIF18B, KIF4A, NUSAP1, KIF14, BIRC5, KIF2C, NEK2, KIF20A, AURKA | 0.001519 | |
| GO:0015630~microtubule cytoskeleton | 7 | 2.11E-04 | TPX2, PRC1, TIMELESS, KIF2C, CDC7, AURKA, MCM2 | 0.002171 | |
| GO:0005829~cytosol | 37 | 3.13E-04 | MCM7, HPGD, GMNN, GMPS, KIF14, NCAPG, ALOX12, HMMR, TYMS, SMC4, AURKA, CDC20, GYS2, CCNB1, NDN, KNTC1, NEK2, ECT2, GTSE1, RRM2, JUP, CDKN2A, UBE2C, SORBS1, ZWINT, DHFR, AR, TPX2, CENPF, PRC1, CENPI, KIF4A, SMS, CDK1, BIRC5, CENPN, KIF2C | 0.002941 | |
| GO:0005871~kinesin complex | 5 | 3.25E-04 | KIF18B, KIF4A, KIF14, KIF2C, KIF20A | 0.002941 | |
| GO:0045120~pronucleus | 3 | 7.84E-04 | CENPF, EZH2, AURKA | 0.006356 | |
| GO:0016020~membrane | 26 | 0.001825 | FEN1, MCM7, NCAPG2, KIF14, NCAPG, TTK, ALOX12, HMMR, ATP2C1, CCNB1, SLIT2, GTSE1, FANCI, KRT2, KRT1, EMP1, ESR1, CRNN, MELK, KIF4A, MCM3, CDK1, MCM4, MCM5, KIF2C, ATP13A3 | 0.014051 | |
| GO:0072686~mitotic spindle | 4 | 0.002084 | SPAG5, CDK1, ECT2, AURKA | 0.015286 | |
| **KEGG Pathway** | | | | |  |
| **Term** | **Count** | **PValue** | **cDEGs** | |  |
| DNA replication(hsa03030) | 9 | 7.97E-11 | RFC5, FEN1, RFC4, MCM7, MCM3, MCM4, MCM5, MCM6, MCM2 | |  |
| Cell cycle (hsa04110) | 12 | 5.37E-10 | CDC20, CCNB1, MCM7, CDKN2A, CDK1, MCM3, MCM4, TTK, CDC7, MCM5, MCM6, MCM2 | |  |
| p53 signaling pathway (hsa04115) | 5 | 0.001159 | CCNB1, RRM2, CDKN2A, CDK1, GTSE1 | |  |
| Oocyte meiosis (hsa04114) | 5 | 0.00724 | CDC20, AR, CCNB1, CDK1, AURKA | |  |
| Fanconi anemia pathway (hsa03460:) | 3 | 0.04857 | FANCI, BRCA1, FANCG | |  |

**Table S5:** Gene-Drug interactions analysis based on our proposed hHubGs using DGIdb database.

| **Gene name** | **Drug name** | **Interaction types** | **Sources** | **PMIDs** |
| --- | --- | --- | --- | --- |
| CDK2 | SU-9516 | inhibitor | ChemblInteractions | 10592235\|11752352\|17139284\|17016423 |
| CDK2 | ALVOCIDIB | inhibitor | MyCancerGenome\|TdgClinicalTrial\|ChemblInteractions | 18465538\|11752352 |
| CDK2 | BMS-387032 | inhibitor | TdgClinicalTrial\|ChemblInteractions\|CancerCommons\|TTD |  |
| CDK2 | AT-7519 | inhibitor | DTC\|TdgClinicalTrial\|ChemblInteractions\|TTD | 10592235\|18656911 |
| CDK2 | AZD-5438 | inhibitor | ChemblInteractions\|TTD |  |
| CDK2 | DINACICLIB | inhibitor | DTC\|MyCancerGenome\|ClearityFoundationClinicalTrial\|ChemblInteractions\|CancerCommons\|MyCancerGenomeClinicalTrial | 23600925 |
| CDK2 | ZOTIRACICLIB | inhibitor | DTC\|ChemblInteractions\|TTD | 22148278 |
| CDK2 | MILCICLIB | inhibitor | DTC\|ChemblInteractions | 19603809 |
| CDK2 | RGB-286638 | inhibitor | ChemblInteractions\|TTD |  |
| CDK2 | PHA-793887 | inhibitor | ChemblInteractions\|TTD |  |
| CDK2 | CYC-065 | inhibitor | TTD |  |
| CDK2 | AG-24322 | inhibitor | ChemblInteractions\|TTD |  |
| CDK2 | RG-547 | inhibitor | ChemblInteractions | 10592235 |
| CDK2 | RONICICLIB | inhibitor | ChemblInteractions |  |
| CDK2 | SELICICLIB | inhibitor | DTC\|MyCancerGenome\|TdgClinicalTrial\|ChemblInteractions\|TTD | 17108108\|21080703 |
| CDK2 | UCN-01 | inhibitor | ChemblInteractions |  |
| CDK2 | OLOMOUCINE | inhibitor | DTC | 10592235\|11752352\|17139284\|17016423 |
| CDK2 | LY-2090314 |  | DTC |  |
| CDK2 | LAPACHONE |  | NCI | 12689523 |
| CDK2 | SP-600125 |  | DTC |  |
| CDK2 | CHEMBL379975 |  | DTC |  |
| CDK2 | (RS)-ROSCOVITINE |  | DTC |  |
| CDK2 | RALTITREXED |  | NCI | 10047461 |
| CDK2 | CHEMBL541400 |  | DTC |  |
| CDK2 | CHEMBL402915 |  | DTC | 18063365 |
| CDK2 | RG-1530 |  | DTC |  |
| CDK2 | AZD-1080 |  | DTC |  |
| CDK2 | CHIR-99021 |  | DTC |  |
| CDK2 | BMS-345541 |  | DTC |  |
| CDK2 | GS 6201 |  | DTC |  |
| CDK2 | CHEMBL225519 |  | DTC |  |
| CDK2 | CHEMBL365617 |  | DTC |  |
| CDK2 | CENISERTIB |  | DTC |  |
| CDK2 | ERIBULIN |  | CIViC | 26006067 |
| CDK2 | TAE-684 |  | DTC |  |
| CDK2 | CHEMBL578061 |  | DTC |  |
| CDK2 | PF-00562271 |  | DTC |  |
| CDK2 | DEXAMETHASONE |  | NCI | 10867026 |
| CDK2 | CHEMBL210618 |  | DTC |  |
| CDK2 | ANDROSTANOLONE |  | NCI | 10698512 |
| CDK2 | CHEMBL557670 |  | DTC | 19410453 |
| CDK2 | CHEMBL373822 |  | DTC | 17181166 |
| CDK2 | R-406 |  | DTC |  |
| CDK2 | KENPAULLONE |  | DTC |  |
| CDK2 | INDOLE-3-CARBINOL |  | NCI | 15611077 |
| CDK2 | AZAKENPAULLONE |  | DTC |  |
| CDK2 | ALSTERPAULLONE |  | DTC |  |
| CDK2 | GW441756X |  | DTC |  |
| CDK2 | GO-6976 |  | DTC |  |
| CDK2 | ACETAMINOPHEN |  | NCI | 14644624 |
| CDK2 | CYC-116 |  | DTC |  |
| CDK2 | CHEMBL259850 |  | DTC |  |
| CDK2 | PACLITAXEL |  | NCI | 16020661 |
| CDK2 | SOTRASTAURIN |  | DTC |  |
| CDK2 | LAUROGUADINE |  | DTC |  |
| CDK2 | CARBOPLATIN |  | CIViC | 26006067 |
| CDK2 | RESVERATROL |  | NCI | 15122319 |
| CDK2 | LOVASTATIN |  | NCI | 9553123 |
| CDK2 | O-CHLOROACETYLCARBAMOYLFUMAGILLOL |  | NCI | 8012959 |
| CDK2 | CHEMBL494107 |  | DTC | 17643111 |
| CDK2 | GSK-269962A |  | DTC |  |
| CHEK1 | SCH-900776 | inhibitor | ChemblInteractions\|TTD |  |
| CHEK1 | LY-2606368 | inhibitor | ChemblInteractions |  |
| CHEK1 | PREXASERTIB | inhibitor | ChemblInteractions\|CIViC | 28490518 |
| CHEK1 | AZD-7762 | inhibitor | TALC\|ChemblInteractions\|TTD |  |
| CHEK1 | RABUSERTIB | inhibitor | ChemblInteractions |  |
| CHEK1 | PF-00477736 | inhibitor | ChemblInteractions |  |
| CHEK1 | XL-844 | inhibitor | ChemblInteractions |  |
| CHEK1 | UCN-01 | inhibitor | ChemblInteractions\|NCI\|TTD | 12244092 |
| CHEK1 | RG-7602 | inhibitor | ChemblInteractions\|TTD |  |
| CHEK1 | CHEMBL2203843 | inhibitor | DTC | 23082860 |
| CHEK1 | RG-7741 | inhibitor | ChemblInteractions |  |
| CHEK1 | DACTOLISIB |  | DTC |  |
| CHEK1 | DOVITINIB |  | DTC |  |
| CHEK1 | CHEMBL506048 |  | DTC | 17978180 |
| CHEK1 | PALBOCICLIB |  | DTC |  |
| CHEK1 | TAE-684 |  | DTC |  |
| CHEK1 | R-406 |  | DTC |  |
| CHEK1 | RG-1530 |  | DTC |  |
| CHEK1 | CHEMBL1997335 |  | DTC |  |
| CHEK1 | KENPAULLONE |  | DTC |  |
| CHEK1 | SP-600125 |  | DTC |  |
| CHEK1 | CHEMBL578061 |  | DTC |  |
| CHEK1 | CYC-116 |  | DTC |  |
| CHEK1 | WITHAFERIN A |  | DTC | 24079846 |
| CHEK1 | CENISERTIB |  | DTC |  |
| CHEK1 | ETOPOSIDE |  | DTC | 22364746 |
| CHEK1 | CI-1040 |  | TTD |  |
| CHEK1 | GEMCITABINE |  | NCI | 17245119 |
| CHEK1 | CISPLATIN |  | CIViC | 28490518 |
| CHEK1 | OLAPARIB |  | CIViC | 28490518 |
| CHEK1 | THYMIDINE |  | NCI | 16280359 |
| CHEK1 | ADRIAMYCIN |  | NCI | 15707569 |
| CHEK1 | DIMETHYLADENINE |  | DTC |  |
| TOP2A | AMRUBICIN | inhibitor | ChemblInteractions | 17628745 |
| TOP2A | EPIRUBICIN | inhibitor | TdgClinicalTrial\|TEND\|PharmGKB | 14728934\|16234514\|17639997 |
| TOP2A | DAUNORUBICIN | inhibitor | DTC\|TdgClinicalTrial\|NCI | 22260166\|1963303\|6380596\|9494516 |
| TOP2A | C-1311 | inhibitor | TdgClinicalTrial\|ChemblInteractions |  |
| TOP2A | MITOXANTRONE | inhibitor | TdgClinicalTrial\|NCI\|TEND | 10451375\|11004693\|18687447\|11752352\|9631585\|9494516\|11278845\|9426516 |
| TOP2A | DEXRAZOXANE | inhibitor | NCI | 11179439\|17652819\|10194547\|11046078\|12911317\|17115008\|11752352\|11984069\|11332155 |
| TOP2A | VOSAROXIN | inhibitor | TdgClinicalTrial\|ChemblInteractions |  |
| TOP2A | DAUNORUBICIN CITRATE | inhibitor | ChemblInteractions |  |
| TOP2A | DOXORUBICIN | inhibitor | DTC\|TdgClinicalTrial\|ClearityFoundationClinicalTrial\|TEND | 21388138\|17016621\|17578914\|17010609\|17351394\|26211460\|11752352\|20170164\|17089011\|22276998 |
| TOP2A | ETOPOSIDE | inhibitor | DTC\|TdgClinicalTrial\|ChemblInteractions\|NCI\|TEND | 25466187\|20006518\|18258442\|8823806\|22867019\|25240702\|26291037\|25003995\|26216018\|26292628\|23360284\|16271071\|23920485\|21435753\|22867097\|16759114\|11678653\|19386396\|24931277\|23566520\|17361331\|25922181\|25941559\|24507920\|24775914\|9485461\|23353750\|25815139\|16309315\|24012683\|19691293\|25800514\|21644529\|22620261\|25945730\|24334150\|17514873\|8870683\|23711769\|11752352\|20863598\|24095018\|26264845\|25799376\|22364746\|16377807\|9494516\|23968711\|18816045\|24326278\|19783445\|9426516 |
| TOP2A | GANCOTAMAB | inhibitor | ChemblInteractions |  |
| TOP2A | PODOFILOX | inhibitor | TdgClinicalTrial\|TEND | 16061385\|1334447\|10783066\|11752352\|1845848\|1331331 |
| TOP2A | AMSACRINE | inhibitor | DTC\|NCI | 1322791\|8823806\|10691026\|8519659\|19155103\|22537681\|17911018\|8632768\|19725581\|11006484\|11716434\|11752352\|25626146\|11473732\|1311390 |
| TOP2A | BERUBICIN HYDROCHLORIDE | inhibitor | ChemblInteractions |  |
| TOP2A | VALRUBICIN | inhibitor | TdgClinicalTrial\|ChemblInteractions\|TEND | 11752352\|16019763 |
| TOP2A | ETOPOSIDE PHOSPHATE | inhibitor | ChemblInteractions |  |
| TOP2A | TENIPOSIDE | inhibitor | TdgClinicalTrial\|ChemblInteractions\|NCI\|TEND | 8702194\|16271071\|17361331\|17514873\|11752352\|16480143\|9426516 |
| TOP2A | IDARUBICIN | inhibitor | TdgClinicalTrial\|TEND | 10203104\|8036155\|12034365\|11836027\|11752352\|10523799 |
| TOP2A | IDARUBICIN HYDROCHLORIDE | inhibitor | ChemblInteractions |  |
| TOP2A | MITOXANTRONE HYDROCHLORIDE | inhibitor | ChemblInteractions |  |
| TOP2A | AMRUBICIN HYDROCHLORIDE | inhibitor | ChemblInteractions |  |
| TOP2A | ALDOXORUBICIN | inhibitor | TdgClinicalTrial\|ChemblInteractions | 25312684 |
| TOP2A | DOXORUBICIN HYDROCHLORIDE | inhibitor | ChemblInteractions |  |
| TOP2A | BECATECARIN | inhibitor | TdgClinicalTrial\|ChemblInteractions |  |
| TOP2A | DAUNORUBICIN HYDROCHLORIDE | inhibitor | ChemblInteractions |  |
| TOP2A | ELLIPTECINE |  | DTC | 18816045\|19783445 |
| TOP2A | CHEMBL607534 |  | DTC | 19783445 |
| TOP2A | DEMETHYLZEYLASTERONE |  | DTC | 11678653 |
| TOP2A | CHEMBL594695 |  | DTC | 19783445 |
| TOP2A | MAKALUVAMINE F |  | DTC | 8691207 |
| TOP2A | ADRIAMYCIN |  | NCI | 9494516 |
| TOP2A | BETULIN |  | DTC | 11754608 |
| TOP2A | CHEMBL1773343 |  | DTC | 21489802 |
| TOP2A | CHEMBL1080077 |  | DTC | 19691293 |
| TOP2A | CARINATIN G |  | DTC |  |
| TOP2A | CHEMBL594257 |  | DTC | 19783445 |
| TOP2A | CHEMBL594153 |  | DTC | 19783445 |
| TOP2A | MAKALUVAMINE A |  | DTC | 8691207 |
| TOP2A | UNGEREMINE |  | DTC | 22014547 |
| TOP2A | CHEMBL2171812 |  | DTC | 22867019 |
| TOP2A | FISETIN |  | DTC | 8759170 |
| TOP2A | KAEMPFERITRIN |  | DTC | 22014228 |
| TOP2A | CHEMBL2332126 |  | DTC |  |
| TOP2A | FRANGULIN B |  | DTC | 20561793 |
| TOP2A | CHEMBL594259 |  | DTC | 19783445 |
| TOP2A | DIAZIRINE |  | DTC | 20006518 |
| TOP2A | ELINAFIDE |  | DTC | 21880496 |
| TOP2A | CHEMBL507986 |  | DTC | 21391686 |
| TOP2A | CHEMBL2023733 |  | DTC | 22276998 |
| TOP2A | FLUOROURACIL |  | PharmGKB |  |
| TOP2A | QUERCETIN |  | DTC | 8759170\|7769390 |
| TOP2A | 4'-O-ACETYLPATENTIFLORIN B |  | DTC | 22119124 |
| TOP2A | DIPHYLLIN |  | DTC | 22119124 |
| TOP2A | MAKALUVAMINE C TFA SALT |  | DTC | 8691207 |
| TOP2A | CHEMBL244268 |  | DTC | 17658777 |
| TOP2A | MAKALUVAMINE E |  | DTC | 8691207 |
| TOP2A | 13-DEOXYDOXORUBICIN |  | TdgClinicalTrial |  |
| TOP2A | DIGITOXIN |  | DTC | 16309315 |
| TOP2A | CHEMBL2171781 |  | DTC | 22867019 |
| TOP2A | VINCRISTINE |  | NCI | 9494516 |
| TOP2A | AMONAFIDE |  | DTC\|TdgClinicalTrial | 24054489\|23353750\|17658777\|26211460\|20170164 |
| TOP2A | CHEMBL596082 |  | DTC | 19783445 |
| TOP2A | LUPEOL |  | DTC | 11754608 |
| TOP2A | CHEMBL2332127 |  | DTC |  |
| TOP2A | CHEMBL594379 |  | DTC | 19783445 |
| TOP2A | CHEMBL2332128 |  | DTC |  |
| TOP2A | HURATOXIN |  | DTC | 24931277 |
| TOP2A | CHEMBL593570 |  | DTC | 19783445 |
| TOP2A | IDRONOXIL |  | ClearityFoundationClinicalTrial |  |
| TOP2A | SECAUBRYOLIDE |  | DTC |  |
| TOP2A | LYCOBETAINE |  | DTC | 23266176 |
| TOP2A | SIMOCYCLINONE D8 |  | DTC | 22867097 |
| TOP2A | CAMPTOTHECIN |  | NCI | 11205246 |
| TOP2A | GENISTEIN |  | DTC | 17139284\|7769390\|17016423 |
| TOP2A | MYRICETIN |  | DTC | 8759170\|7769390 |
| TOP2A | HYDROQUINONE |  | NCI | 15833037 |
| TOP2A | OLEANDEROLIDE |  | DTC | 24326278 |
| TOP2A | PACLITAXEL |  | TdgClinicalTrial |  |
| TOP2A | CHEMBL2171794 |  | DTC | 22867019 |
| TOP2A | TRICITRINOL B |  | DTC | 21761866 |
| CDK1 | AG-24322 | inhibitor | ChemblInteractions\|TTD |  |
| CDK1 | RONICICLIB | inhibitor | ChemblInteractions |  |
| CDK1 | AT-7519 | inhibitor | DTC\|ChemblInteractions | 18656911 |
| CDK1 | DINACICLIB | inhibitor | MyCancerGenome\|ClearityFoundationClinicalTrial\|ChemblInteractions\|CancerCommons\|MyCancerGenomeClinicalTrial |  |
| CDK1 | ZOTIRACICLIB | inhibitor | ChemblInteractions |  |
| CDK1 | ALVOCIDIB | inhibitor | MyCancerGenome\|TdgClinicalTrial\|ChemblInteractions | 11752352 |
| CDK1 | CHIR-99021 | inhibitor | TTD |  |
| CDK1 | RIVICICLIB | inhibitor | ChemblInteractions\|TTD |  |
| CDK1 | SELICICLIB | inhibitor | ChemblInteractions\|TTD | 17108108 |
| CDK1 | ALSTERPAULLONE | inhibitor | DTC | 11752352 |
| CDK1 | PHA-793887 | inhibitor | ChemblInteractions\|TTD |  |
| CDK1 | RGB-286638 | inhibitor | ChemblInteractions\|TTD |  |
| CDK1 | MILCICLIB | inhibitor | ChemblInteractions |  |
| CDK1 | AZD-5438 | inhibitor | ChemblInteractions |  |
| CDK1 | RG-547 | inhibitor | ChemblInteractions |  |
| CDK1 | KENPAULLONE |  | TTD |  |
| CDK1 | CLOFIBRATE |  | DTC | 16680159 |
| CDK1 | SP-600125 |  | DTC |  |
| CDK1 | CHEMBL1082552 |  | DTC | 16680159 |
| CDK1 | FENOFIBRATE |  | DTC | 16680159 |
| CDK1 | SOTRASTAURIN |  | DTC |  |
| CDK1 | GO-6976 |  | DTC |  |
| CDK1 | LAUROGUADINE |  | DTC |  |
| CDK1 | BMS-345541 |  | DTC |  |
| CDK1 | PATULIN |  | DTC | 16680159 |
| CDK1 | SERTRALINE |  | DTC | 16680159 |
| CDK1 | GW441756X |  | DTC |  |
| CDK1 | CHEMBL403183 |  | DTC | 16680159 |
| CDK1 | CENISERTIB |  | DTC |  |
| CDK1 | ARUNCIN B |  | DTC | 22197393 |
| CDK1 | LY-2090314 |  | DTC |  |
| CDK1 | PROTUBOXEPIN A |  | DTC | 22595423 |
| CDK1 | CHEMBL541400 |  | DTC |  |
| CDK1 | AZD-1080 |  | DTC |  |
| CDK1 | CHEMBL225519 |  | DTC |  |
| CDK1 | R-406 |  | DTC |  |
| CDK1 | CYC-116 |  | DTC |  |
| CDK1 | ROTENONE |  | DTC | 16680159 |
| CDK1 | SNS-314 |  | DTC |  |
| CDK1 | PF-00562271 |  | DTC |  |
| CDK1 | CLOTRIMAZOLE |  | DTC | 16680159 |
| CDK1 | WITHAFERIN A |  | DTC | 24079846 |
| CDK1 | CINNARIZINE |  | DTC | 16680159 |
| CDK1 | RG-1530 |  | DTC |  |
| CDK1 | CHEMBL578061 |  | DTC |  |
| CDK1 | (RS)-ROSCOVITINE |  | DTC |  |
| CDK1 | RUCAPARIB |  | DTC |  |
| CDK1 | TAE-684 |  | DTC |  |
| BRCA1 | CHEMBL1481721 |  | DTC |  |
| BRCA1 | DAIDZIN |  | DTC |  |
| BRCA1 | CHEMBL66279 |  | DTC |  |
| BRCA1 | CHEMBL114544 |  | DTC |  |
| BRCA1 | CARBOPLATIN |  | ClearityFoundationBiomarkers\|JAX-CKB\|CIViC | 25847936\|25824335\|21135055\|27998224 |
| BRCA1 | CHEMBL515763 |  | DTC |  |
| BRCA1 | IROFULVEN |  | NCI | 17229870 |
| BRCA1 | GLABRIDIN |  | DTC |  |
| BRCA1 | CHEMBL546649 |  | DTC |  |
| BRCA1 | CHEMBL1998001 |  | DTC |  |
| BRCA1 | CHEMBL1405979 |  | DTC |  |
| BRCA1 | PIPERINE |  | DTC |  |
| BRCA1 | CHEMBL1214407 |  | DTC |  |
| BRCA1 | CHEMBL1308677 |  | DTC |  |
| BRCA1 | DENOSUMAB |  | JAX-CKB | 27322743 |
| BRCA1 | TANSHINONE IIA SULFONATE |  | DTC |  |
| BRCA1 | RUCAPARIB |  | ClearityFoundationBiomarkers\|JAX-CKB\|CIViC\|PharmGKB\|OncoKB | 28588062\|27908594\|26779812\|27002934\|27454289 |
| BRCA1 | CHEMBL1333798 |  | DTC |  |
| BRCA1 | RESVERATROL |  | DTC |  |
| BRCA1 | QUINALIZARIN |  | DTC |  |
| BRCA1 | CHEMBL323356 |  | DTC |  |
| BRCA1 | PHENAMIL METHANESULFONATE |  | DTC |  |
| BRCA1 | NIRAPARIB |  | ClearityFoundationBiomarkers\|JAX-CKB\|PharmGKB\|OncoKB | 27717299\|23810788 |
| BRCA1 | GAMMA-FAGARINE |  | DTC |  |
| BRCA1 | CHEMBL12658 |  | DTC |  |
| BRCA1 | CALYCOSIN |  | DTC |  |
| BRCA1 | DAUNORUBICIN HYDROCHLORIDE |  | DTC |  |
| BRCA1 | 7-HYDROXY ISOFLAVONE |  | DTC |  |
| BRCA1 | CHEMBL1320798 |  | DTC |  |
| BRCA1 | PIFEXOLE |  | DTC |  |
| BRCA1 | OLAPARIB |  | ClearityFoundationBiomarkers\|JAX-CKB\|CIViC\|PharmGKB\|OncoKB | 31157963\|23346317\|26546619\|20609467\|27454287\|25193512\|30345884\|25366685\|19553641\|28792849\|25218906\|28578601\|21862407\|22172724\|30797618\|31538027\|24882434 |
| BRCA1 | CHEMBL316796 |  | DTC |  |
| BRCA1 | CHEMBL587620 |  | DTC |  |
| BRCA1 | NILE RED |  | DTC |  |
| BRCA1 | DIMETHYL YELLOW |  | DTC |  |
| BRCA1 | PAMIPARIB |  | JAX-CKB |  |
| BRCA1 | CHLORAMBUCIL |  | JAX-CKB | 25193512 |
| BRCA1 | CHEMBL589207 |  | DTC |  |
| BRCA1 | EVEROLIMUS |  | JAX-CKB | 26546619 |
| BRCA1 | CHEMBL446567 |  | DTC |  |
| BRCA1 | CYCLOPHOSPHAMIDE |  | JAX-CKB | 25589624 |
| BRCA1 | CHEMBL588525 |  | DTC |  |
| BRCA1 | BLEOMYCIN |  | NCI | 14559807 |
| BRCA1 | CHEMBL1404865 |  | DTC |  |
| BRCA1 | DEPHOSTATIN |  | DTC |  |
| BRCA1 | CHEMBL1709259 |  | DTC |  |
| BRCA1 | ACRIFLAVINE |  | DTC |  |
| BRCA1 | TIAPROFENIC ACID |  | DTC |  |
| BRCA1 | QUARFLOXIN |  | CIViC | 28211448 |
| BRCA1 | CHEMBL581929 |  | DTC |  |
| BRCA1 | CHEMBL546865 |  | DTC |  |
| BRCA1 | ADAVOSERTIB |  | JAX-CKB | 27998224 |
| BRCA1 | 2-PHENYLQUINOLINE |  | DTC |  |
| BRCA1 | TEMOZOLOMIDE |  | JAX-CKB |  |
| BRCA1 | TAMOXIFEN |  | NCI\|PharmGKB | 11130383\|15197194\|16331614\|15750629\|16636335 |
| BRCA1 | MITOXANTRONE |  | NCI | 12684687 |
| BRCA1 | CHEMBL1574420 |  | DTC |  |
| BRCA1 | VINORELBINE |  | JAX-CKB\|NCI | 26801247\|14559807 |
| BRCA1 | CEDIRANIB |  | CIViC | 25218906 |
| BRCA1 | AURINTRICARBOXYLIC ACID |  | DTC |  |
| BRCA1 | CHEMBL588683 |  | DTC |  |
| BRCA1 | 7-HYDROXYFLAVONE |  | DTC |  |
| BRCA1 | HARMALOL |  | DTC |  |
| BRCA1 | CHEMBL273891 |  | DTC |  |
| BRCA1 | VELIPARIB |  | ClearityFoundationBiomarkers\|JAX-CKB\|CIViC | 29338080\|26801247\|25824335\|25589624\|26842236 |
| BRCA1 | CHEMBL484663 |  | DTC |  |
| BRCA1 | DOXORUBICIN HYDROCHLORIDE |  | DTC |  |
| BRCA1 | PURPURIN |  | DTC |  |
| BRCA1 | 6-HYDROXYFLAVONE |  | DTC |  |
| BRCA1 | PACLITAXEL |  | JAX-CKB\|NCI | 12684687 |
| BRCA1 | OXALIPLATIN |  | ClearityFoundationBiomarkers\|CIViC | 25072261 |
| BRCA1 | CHEMBL1078244 |  | DTC |  |
| BRCA1 | THUNBERGINOL E |  | DTC |  |
| BRCA1 | CHRYSIN DIMETHYL ETHER |  | DTC |  |
| BRCA1 | 2X-121 |  | ClearityFoundationBiomarkers\|JAX-CKB | 26513298 |
| BRCA1 | RIBOFLAVIN |  | DTC |  |
| BRCA1 | CISPLATIN |  | ClearityFoundationBiomarkers\|JAX-CKB\|NCI\|CIViC | 29338080\|25847936\|25193512\|26801247\|16982732\|25072261\|27454289 |
| BRCA1 | APIGENIN |  | DTC |  |
| BRCA1 | CHEMBL581910 |  | DTC |  |
| BRCA1 | TALAZOPARIB |  | JAX-CKB\|CIViC\|PharmGKB\|OncoKB | 26546619\|23881923\|28242752 |
| BRCA1 | EMODIN |  | NCI | 14694444 |
| BRCA1 | CHEMBL600315 |  | DTC |  |
| BRCA1 | DOXORUBICIN |  | NCI | 12698198 |
| BRCA1 | ONONIN |  | DTC |  |
| BRCA1 | SEMUSTINE |  | DTC |  |
| BRCA1 | THIABENDAZOLE |  | DTC |  |
| BRCA1 | CHEMBL1420181 |  | DTC |  |
| BRCA1 | GEMCITABINE |  | NCI\|CIViC | 29338080\|12684687 |
| BRCA1 | DIPYRIDAMOLE |  | DTC |  |
| BRCA1 | VITAMIN B2 |  | DTC |  |
| BRCA1 | IRINOTECAN |  | JAX-CKB | 26842236 |
| BRCA1 | CHEMBL593254 |  | DTC |  |
| BRCA1 | CHEMBL452954 |  | DTC |  |
| BRCA1 | WISTIN |  | DTC |  |
| BRCA1 | CHEMBL153505 |  | DTC |  |
| BRCA1 | ILEPCIMIDE |  | DTC |  |
| BRCA1 | BERZOSERTIB |  | JAX-CKB |  |
| PLK1 | BI-2536 | inhibitor | TALC\|DTC\|ChemblInteractions | 25685941 |
| PLK1 | ONVANSERTIB | inhibitor | TTD |  |
| PLK1 | GSK-461364 | inhibitor | TALC\|MyCancerGenome\|ChemblInteractions\|TTD |  |
| PLK1 | TAK-960 | inhibitor | ChemblInteractions\|TTD |  |
| PLK1 | CAFUSERTIB | inhibitor | ChemblInteractions |  |
| PLK1 | VOLASERTIB | inhibitor | TALC\|MyCancerGenome\|ChemblInteractions\|TTD |  |
| PLK1 | NMS-1286937 | inhibitor | MyCancerGenome\|ChemblInteractions\|TTD |  |
| PLK1 | MK-1496 | inhibitor | ChemblInteractions\|TTD |  |
| PLK1 | HMN-214 | inhibitor | ChemblInteractions |  |
| PLK1 | CHEMBL116548 |  | DTC |  |
| PLK1 | OLEIC ACID |  | DTC |  |
| PLK1 | AMORFRUTIN A |  | DTC |  |
| PLK1 | CHEMBL1362503 |  | DTC |  |
| PLK1 | LOBARIC ACID |  | DTC |  |
| PLK1 | CHEMBL118378 |  | DTC |  |
| PLK1 | CHEMBL585628 |  | DTC |  |
| PLK1 | CHEMBL1310138 |  | DTC |  |
| PLK1 | 3,5-DIPHENYLISOXAZOLE |  | DTC |  |
| PLK1 | CHEMBL1363249 |  | DTC |  |
| PLK1 | OLEOYL DOPAMINE |  | DTC |  |
| PLK1 | DIPYRIDAMOLE |  | DTC |  |
| PLK1 | CHEMBL24983 |  | DTC |  |
| PLK1 | CHEMBL421431 |  | DTC |  |
| PLK1 | GUTHION |  | DTC |  |
| PLK1 | CHEMBL225519 |  | DTC |  |
| PLK1 | GW843682X |  | DTC |  |
| PLK1 | FLUORESCEIN DIACETATE |  | DTC |  |
| PLK1 | HYDROXYZINE PAMOATE |  | DTC |  |
| PLK1 | PROTOPORPHYRIN |  | DTC |  |
| PLK1 | 1,4-DIMETHOXYANTHRAQUINONE |  | DTC |  |
| PLK1 | CHEMBL528373 |  | DTC |  |
| PLK1 | PHOSMET |  | DTC |  |
| PLK1 | RIGOSERTIB |  | TdgClinicalTrial\|TTD |  |
| PLK1 | 6-CHLORONICOTINIC ACID |  | DTC |  |
| PLK1 | PYRONIN Y |  | DTC |  |
| PLK1 | CHEMBL599098 |  | DTC |  |
| PLK1 | THIMEROSAL |  | DTC |  |
| PLK1 | LANSOPRAZOLE |  | DTC |  |
| PLK1 | CEFSULODIN SODIUM |  | DTC |  |
| PLK1 | ACID BLUE 129 |  | DTC |  |
| PLK1 | CHEMBL584619 |  | DTC |  |
| PLK1 | CHEMBL388979 |  | DTC |  |
| PLK1 | GYROPHORIC ACID |  | DTC |  |
| PLK1 | CEFACLOR |  | DTC |  |
| PLK1 | CHEMBL538798 |  | DTC |  |
| PLK1 | CHEMBL118678 |  | DTC |  |
| PLK1 | CHEMBL121556 |  | DTC |  |
| PLK1 | CHEMBL1503729 |  | DTC |  |
| PLK1 | CHEMBL429095 |  | DTC |  |
| PLK1 | CHEMBL1451216 |  | DTC |  |
| PLK1 | PURPURIN |  | DTC |  |
| PLK1 | CHEMBL122355 |  | DTC |  |
| PLK1 | SOTRASTAURIN |  | DTC |  |
| PLK1 | FLUPIRTINE MALEATE |  | DTC |  |
| PLK1 | CHEMBL602969 |  | DTC |  |
| PLK1 | CHEMBL467987 |  | DTC |  |
| PLK1 | CHEMBL1242101 |  | TTD |  |
| PLK1 | CHEMBL527584 |  | DTC |  |
| PLK1 | BENSERAZIDE HYDROCHLORIDE |  | DTC |  |
| PLK1 | CHEMBL171699 |  | DTC |  |
| PLK1 | CHEMBL1605629 |  | DTC |  |
| PLK1 | CHEMBL1224755 |  | DTC |  |
| PLK1 | WORTMANNIN |  | NCI | 17135248\|10592235 |
| PLK1 | SCLEROTIORIN |  | DTC |  |
| PLK1 | ALPHAPRODINE HYDROCHLORIDE |  | DTC |  |
| PLK1 | CHEMBL599924 |  | DTC |  |
| PLK1 | TOPOTECAN HYDROCHLORIDE |  | DTC |  |
| PLK1 | EMBELIN |  | DTC |  |
| PLK1 | 2,4-DIHYDROXYBENZOPHENONE |  | DTC |  |
| PLK1 | DISULFIRAM |  | DTC |  |
| PLK1 | CHEMBL151797 |  | DTC |  |
| PLK1 | CHEMBL1412002 |  | DTC |  |
| PLK1 | PENTOSALEN |  | DTC |  |
| PLK1 | CHEMBL297304 |  | DTC |  |
| PLK1 | PYROGALLOL RED |  | DTC |  |
| PLK1 | SENNOSIDE B |  | DTC |  |
| PLK1 | SIMVASTATIN |  | DTC |  |
| PLK1 | CHEMBL223575 |  | DTC |  |
| PLK1 | CHEMBL599957 |  | DTC |  |
| PLK1 | CHEMBL565893 |  | DTC |  |
| PLK1 | CHEMBL505670 |  | DTC |  |
| PLK1 | CHEMBL125044 |  | DTC |  |
| PLK1 | IDARUBICIN HYDROCHLORIDE |  | DTC |  |
| PLK1 | CHEMBL599712 |  | DTC |  |
| PLK1 | CHEMBL164449 |  | DTC |  |
| PLK1 | CHEMBL592124 |  | DTC |  |
| PLK1 | CHEMBL1310701 |  | DTC |  |
| PLK1 | CHEMBL585654 |  | DTC |  |
| PLK1 | CHEMBL515505 |  | DTC |  |
| PLK1 | TAE-684 |  | DTC |  |
| PLK1 | PENTACHLOROPHENOL |  | DTC |  |
| PLK1 | CRYPTOTANSHINONE |  | DTC |  |
| PLK1 | CHEMBL184450 |  | DTC |  |
| PLK1 | CHEMBL1707907 |  | DTC |  |
| PLK1 | CHEMBL602807 |  | DTC |  |
| PLK1 | HAEMATOXYLIN |  | DTC |  |
| PLK1 | OMEPRAZOLE |  | DTC |  |
| PLK1 | CHEMBL28721 |  | DTC |  |
| PLK1 | AMBUNOL |  | DTC |  |
| PLK1 | CHEMBL1871992 |  | DTC |  |
| PLK1 | CHEMBL1232076 |  | DTC |  |
| PLK1 | CHEMBL1537417 |  | DTC |  |
| PLK1 | LITHOCHOLIC ACID |  | DTC |  |
| PLK1 | MYRICETIN |  | DTC |  |
| PLK1 | CEFAMANDOLE SODIUM |  | DTC |  |
| PLK1 | CHEMBL534535 |  | DTC |  |
| PLK1 | CHEMBL587449 |  | DTC |  |
| PLK1 | DIFFRACTAIC ACID |  | DTC |  |
| PLK1 | CHEMBL590665 |  | DTC |  |
| PLK1 | RG-1530 |  | DTC |  |
| PLK1 | CHEMBL1486366 |  | DTC |  |
| PLK1 | TOSUFLOXACIN TOSYLATE |  | DTC |  |
| PLK1 | PURPUROGALLIN |  | DTC |  |
| PLK1 | ERYTHROMYCIN |  | DTC |  |
| PLK1 | CHEMBL1426508 |  | DTC |  |
| PLK1 | BENZBROMARONE |  | DTC |  |
| PLK1 | TRITIOZINE |  | DTC |  |
| PLK1 | BETUNOLIC ACID |  | DTC |  |
| PLK1 | OXIDOPAMINE HYDROCHLORIDE |  | DTC |  |
| PLK1 | CHEMBL84281 |  | DTC |  |
| PLK1 | CHEMBL462861 |  | DTC |  |
| PLK1 | PHYSODIC ACID |  | DTC |  |
| PLK1 | INAMRINONE |  | DTC |  |
| PLK1 | CHEMBL119878 |  | DTC |  |
| PLK1 | CHEMBL1287980 |  | DTC |  |
| PLK1 | CHEMBL495852 |  | DTC |  |
| PLK1 | CHEMBL577365 |  | DTC |  |
| PLK1 | OXYTETRACYCLINE |  | DTC |  |
| PLK1 | CHEMBL555260 |  | DTC |  |
| PLK1 | CHEMBL585279 |  | DTC |  |
| PLK1 | OXYPHENBUTAZONE |  | DTC |  |
| PLK1 | CHEMBL535051 |  | DTC |  |
| PLK1 | CEPHALOCHROMIN |  | DTC |  |
| PLK1 | CHEMBL1701915 |  | DTC |  |
| PLK1 | CHEMBL103583 |  | DTC |  |
| PLK1 | CHEMBL592314 |  | DTC |  |
| PLK1 | APOMORPHINE HYDROCHLORIDE HEMIHYDRATE |  | DTC |  |
| PLK1 | CHEMBL599255 |  | DTC |  |
| PLK1 | (4-CHLOROPHENYL)(MORPHOLINO)METHANETHIONE |  | DTC |  |
| PLK1 | CHEMBL1255944 |  | DTC |  |
| PLK1 | CHEMBL1547715 |  | DTC |  |
| PLK1 | ACRIFLAVINE |  | DTC |  |
| PLK1 | IMIDACLOPRID |  | DTC |  |
| PLK1 | CHEMBL1519374 |  | DTC |  |
| PLK1 | HEXACHLOROPHENE |  | DTC |  |
| PLK1 | CHEMBL582764 |  | DTC |  |
| PLK1 | CHEMBL588749 |  | DTC |  |
| PLK1 | GW7647 |  | DTC |  |
| PLK1 | CHEMBL275311 |  | DTC |  |
| PLK1 | 2,6-DIMETHOXYQUINONE |  | DTC |  |
| PLK1 | CHEMBL175266 |  | DTC |  |
| PLK1 | CHEMBL374632 |  | DTC |  |
| PLK1 | CHEMBL530149 |  | DTC |  |
| PLK1 | ELLAGIC ACID |  | DTC |  |
| PLK1 | CHEMBL1489812 |  | DTC |  |
| PLK1 | CHEMBL1459280 |  | DTC |  |
| PLK1 | EBSELEN |  | DTC |  |
| PLK1 | CHEMBL523200 |  | DTC |  |
| PLK1 | PROROXAN HYDROCHLORIDE |  | DTC |  |
| PLK1 | 4-CHLOROMERCURIBENZOIC ACID |  | DTC |  |
| PLK1 | CHEMBL582478 |  | DTC |  |
| PLK1 | VANITIOLIDE |  | DTC |  |
| PLK1 | THIOGUANOSINE |  | DTC |  |
| PLK1 | ACITRETIN |  | DTC |  |
| PLK1 | LOMOFUNGIN |  | DTC |  |
| PLK1 | PENTABROMOPHENOL |  | DTC |  |
| PLK1 | THIRAM |  | DTC |  |
| PLK1 | CHEMBL274921 |  | DTC |  |
| PLK1 | NANAOMYCIN |  | DTC |  |
| PLK1 | CHEMBL1271059 |  | DTC |  |
| PLK1 | SLAZINIC ACID |  | DTC |  |
| PLK1 | CHEMBL1306556 |  | DTC |  |
| PLK1 | CHEMBL548629 |  | DTC |  |
| PLK1 | CHEMBL592558 |  | DTC |  |
| PLK1 | CHLORPYRIFOS |  | DTC |  |
| PLK1 | STAVUDINE |  | DTC |  |
| CCNA2 | GENISTEIN |  | NCI | 9664138 |
| CCNA2 | SELICICLIB |  | DTC | 21080703 |
| CCNA2 | SURAMIN |  | NCI | 10208280 |
| CCNA2 | TAMOXIFEN |  | PharmGKB | 24622579 |
| CCNB1 | SELICICLIB |  | DTC | 21080703 |
| CCNB1 | PROTOAPIGENONE |  | DTC |  |
| CCNB1 | KENPAULLONE |  | TTD |  |
| TYMS | RALTITREXED | inhibitor | TdgClinicalTrial\|TEND\|PharmGKB | 18773878\|10598555\|17203168\|10430100\|10592235\|11752352\|10499608\|10482907\|15353299\|16456808\|10047461\|10496350 |
| TYMS | PEMETREXED | inhibitor | ClearityFoundationBiomarkers\|TdgClinicalTrial\|TEND\|CIViC | 12571811\|15795320\|11524555\|11742712\|12023793\|23645741\|23060591\|14596699\|11763166\|21367480\|11752352\|11531245\|11848474\|11252887\|26502926 |
| TYMS | CAPECITABINE | inhibitor | ClearityFoundationBiomarkers\|ClearityFoundationClinicalTrial\|ChemblInteractions | 15134221\|16926630\|15866500\|15132128\|11752352\|15709193 |
| TYMS | FLOXURIDINE | inhibitor | TdgClinicalTrial\|ClearityFoundationClinicalTrial\|ChemblInteractions\|TEND | 10697523\|10891536\|10697524\|10482907\|10553409 |
| TYMS | FLUOROURACIL | inhibitor\|other/unknown | DTC\|ClearityFoundationClinicalTrial\|ChemblInteractions\|CIViC | 16563096\|20628391\|25544046\|16609021\|16719540\|16596248\|16538493\|11752352\|15353299\|24450514 |
| TYMS | TEGAFUR | inhibitor | TdgClinicalTrial |  |
| TYMS | METHOTREXATE | inhibitor | CIViC | 23652803\|24284432 |
| TYMS | THYMIDINE MONOPHOSPHATE |  | DTC | 6434741\|17139284\|17016423 |
| TYMS | LEUCOVORIN |  | TdgClinicalTrial\|TEND |  |
| TYMS | AZACITIDINE |  | PharmGKB | 29205204 |
| TYMS | PREDNISONE |  | PharmGKB |  |
| TYMS | ETOPOSIDE |  | PharmGKB |  |
| TYMS | ENILURACIL |  | NCI | 10692560 |
| TYMS | OSI-7904 |  | TdgClinicalTrial |  |
| TYMS | TOPOTECAN |  | NCI | 10803925 |
| TYMS | TAMOXIFEN |  | NCI | 9615734 |
| TYMS | HYDROCORTISONE |  | NCI | 2707640 |
| TYMS | VINCRISTINE |  | NCI | 2804079 |
| TYMS | DEOXYURIDINE MONOPHOSPHATE |  | DTC | 6434741\|10592235\|17139284\|17016423 |
| TYMS | CYTARABINE |  | PharmGKB |  |
| TYMS | VERAPAMIL |  | NCI | 3436366 |
| TYMS | RESERPINE |  | NCI | 2866100 |
| TYMS | CISPLATIN |  | NCI | 3337743 |
| TYMS | DEXAMETHASONE |  | NCI\|PharmGKB | 2707640\|3398844 |
| TYMS | CARBOGEN |  | NCI | 12915890 |
| TYMS | PLATINUM |  | PharmGKB | 25677447 |
| TYMS | PHENTOLAMINE |  | NCI | 2866100 |
| TYMS | ARFOLITIXORIN |  | TdgClinicalTrial |  |
| TYMS | DOXIFLURIDINE |  | NCI | 2972339 |
| TYMS | IRINOTECAN |  | CIViC\|PharmGKB | 20628391\|21273624\|16456808 |
| TYMS | 9-AMINOCAMPTOTHECIN |  | NCI | 10803925 |
| TYMS | INDOMETHACIN |  | NCI | 2707640 |
| TYMS | AZATHIOPRINE |  | PharmGKB | 26332308 |
| TYMS | ASPARAGINASE |  | PharmGKB |  |
| TYMS | HYDROXYCHLOROQUINE |  | PharmGKB | 18322994 |
| TYMS | DAUNORUBICIN |  | NCI\|PharmGKB | 2967076 |
| TYMS | SULFASALAZINE |  | PharmGKB | 18322994 |

**Reference**

1. Feng, J. Upregulation of microRNA-4262 targets KaiSO (ZBTB33) to inhibit the proliferation and EMT of cervical cancer cells. *Oncol. Res.* **2018**, *26*, doi:10.3727/096504017X15021536183526.

2. Venkataramnan, S.; Binti Zainol Izam Khan, W.N. CERVICAL CANCER AND GENE EXPRESSION ANALYSIS WITH KEY GENES IDENTIFICATION BY COMPUTATIONAL METHOD. *J. Bio Innov.* **2020**, *9*, doi:10.46344/jbino.2020.v09i05.26.

3. Mei, Y.; Jiang, P.; Shen, N.; Fu, S.; Zhang, J. Identification of miRNA-mRNA Regulatory Network and Construction of Prognostic Signature in Cervical Cancer. *DNA Cell Biol.* **2020**, *39*, doi:10.1089/dna.2020.5452.

4. Liu, J.; Nie, S.; Gao, M.; Jiang, Y.; Wan, Y.; Ma, X.; Zhou, S.; Cheng, W. Identification of EPHX2 and RMI2 as two novel key genes in cervical squamous cell carcinoma by an integrated bioinformatic analysis. *J. Cell. Physiol.* **2019**, *234*, doi:10.1002/jcp.28731.

5. Qiu, H.Z.; Huang, J.; Xiang, C.C.; Li, R.; Zuo, E.D.; Zhang, Y.; Shan, L.; Cheng, X. Screening and Discovery of New Potential Biomarkers and Small Molecule Drugs for Cervical Cancer: A Bioinformatics Analysis. *Technol. Cancer Res. Treat.* **2020**, *19*, doi:10.1177/1533033820980112.

6. Meneur, C.; Eswaran, S.; Adiga, D.; Sriharikrishnaa, S.; Nadeem, K.G.; Mallya, S.; Chakrabarty, S.; Kabekkodu, S.P. Analysis of Nuclear Encoded Mitochondrial Gene Networks in Cervical Cancer. *Asian Pacific J. Cancer Prev.* **2021**, *22*, doi:10.31557/APJCP.2021.22.6.1799.

7. Wu, X.; Peng, L.; Zhang, Y.; Chen, S.; Lei, Q.; Li, G.; Zhang, C. Identification of key genes and pathways in cervical cancer by bioinformatics analysis. *Int. J. Med. Sci.* **2019**, *16*, doi:10.7150/ijms.34172.

8. Mei, J.; Xing, Y.; Lv, J.; Gu, D.; Pan, J.; Zhang, Y.; Liu, J. Construction of an immune-related gene signature for prediction of prognosis in patients with cervical cancer. *Int. Immunopharmacol.* **2020**, *88*, doi:10.1016/j.intimp.2020.106882.

9. Yi, Y.; Fang, Y.; Wu, K.; Liu, Y.; Zhang, W. Comprehensive gene and pathway analysis of cervical cancer progression. *Oncol. Lett.* **2020**, *19*, doi:10.3892/ol.2020.11439.

10. Deng, S.P.; Zhu, L.; Huang, D.S. Predicting Hub Genes Associated with Cervical Cancer through Gene Co-Expression Networks. *IEEE/ACM Trans. Comput. Biol. Bioinforma.* **2016**, *13*, doi:10.1109/TCBB.2015.2476790.

11. Yang, H. ju; Xue, J. min; Li, J.; Wan, L. hong; Zhu, Y. xi Identification of key genes and pathways of diagnosis and prognosis in cervical cancer by bioinformatics analysis. *Mol. Genet. Genomic Med.* **2020**, *8*, doi:10.1002/mgg3.1200.

12. Wang, J.; Zheng, H.; Han, Y.; Wang, G.; Li, Y. A Novel Four-Gene Prognostic Signature as a Risk Biomarker in Cervical Cancer. *Int. J. Genomics* **2020**, *2020*, doi:10.1155/2020/4535820.

13. Liu, J.; Yang, J.; Gao, F.; Li, S.; Nie, S.; Meng, H.; Sun, R.; Wan, Y.; Jiang, Y.; Ma, X.; et al. A microRNA-Messenger RNA Regulatory Network and Its Prognostic Value in Cervical Cancer. *DNA Cell Biol.* **2020**, *39*, doi:10.1089/dna.2020.5590.

14. Ouyang, D.; Ouyang, D.; Yang, P.; Cai, J.; Sun, S.; Wang, Z. Comprehensive analysis of prognostic alternative splicing signature in cervical cancer. *Cancer Cell Int.* **2020**, *20*, doi:10.1186/s12935-020-01299-4.

15. Chen, H.; Wang, X.; Jia, H.; Tao, Y.; Zhou, H.; Wang, M.; Wang, X.; Fang, X. Bioinformatics analysis of key genes and pathways of cervical cancer. *Onco. Targets. Ther.* **2020**, *13*, 13275–13283, doi:10.2147/OTT.S281533.

16. Xue, H.; Sun, Z.; Wu, W.; Du, D.; Liao, S. Identification of hub genes as potential prognostic biomarkers in cervical cancer using comprehensive bioinformatics analysis and validation studies. *Cancer Manag. Res.* **2021**, *13*, doi:10.2147/CMAR.S282989.

17. Zhao, Q.; Li, H.; Zhu, L.; Hu, S.; Xi, X.; Liu, Y.; Liu, J.; Zhong, T. Bioinformatics analysis shows that top2a functions as a key candidate gene in the progression of cervical cancer. *Biomed. Reports* **2020**, *13*, doi:10.3892/br.2020.1328.

18. Ma, X.; Liu, J.; Wang, H.; Jiang, Y.; Wan, Y.; Xia, Y.; Cheng, W. Identification of crucial aberrantly methylated and differentially expressed genes related to cervical cancer using an integrated bioinformatics analysis. *Biosci. Rep.* **2020**, *40*, doi:10.1042/BSR20194365.

19. Mallik, S.; Seth, S.; Bhadra, T.; Zhao, Z. A linear regression and deep learning approach for detecting reliable genetic alterations in cancer using dna methylation and gene expression data. *Genes (Basel).* **2020**, *11*, doi:10.3390/genes11080931.

20. Liu, J.; Li, S.; Lin, L.; Jiang, Y.; Wan, Y.; Zhou, S.; Cheng, W. Co-expression network analysis identified atypical chemokine receptor 1 (ACKR1) association with lymph node metastasis and prognosis in cervical cancer. *Cancer Biomarkers* **2020**, *27*, doi:10.3233/CBM-190533.

21. Tu, S.; Zhang, H.; Yang, X.; Wen, W.; Song, K.; Yu, X.; Qu, X. Screening of cervical cancer-related hub genes based on comprehensive bioinformatics analysis. *Cancer Biomarkers* **2021**, doi:10.3233/cbm-203262.

22. Wu, K.; Yi, Y.; Liu, F.; Wu, W.; Chen, Y.; Zhang, W. Identification of key pathways and genes in the progression of cervical cancer using bioinformatics analysis. *Oncol. Lett.* **2018**, *16*, doi:10.3892/ol.2018.8768.

23. Liu, J.; Wu, Z.; Wang, Y.; Nie, S.; Sun, R.; Yang, J.; Cheng, W. A prognostic signature based on immune-related genes for cervical squamous cell carcinoma and endocervical adenocarcinoma. *Int. Immunopharmacol.* **2020**, *88*, doi:10.1016/j.intimp.2020.106884.

24. Liu, J.; Liu, S.; Yang, X. Construction of Gene Modules and Analysis of Prognostic Biomarkers for Cervical Cancer by Weighted Gene Co-Expression Network Analysis. *Front. Oncol.* **2021**, *11*, doi:10.3389/fonc.2021.542063.

25. Xu, Z.; Zhou, Y.; Shi, F.; Cao, Y.; Dinh, T.L.A.; Wan, J.; Zhao, M. Investigation of differentially-expressed microRNAs and genes in cervical cancer using an integrated bioinformatics analysis. *Oncol. Lett.* **2017**, *13*, doi:10.3892/ol.2017.5766.

26. Liu, Y.; Yi, Y.; Wu, W.; Wu, K.; Zhang, W. Bioinformatics prediction and analysis of hub genes and pathways of three types of gynecological cancer. *Oncol. Lett.* **2019**, *18*, doi:10.3892/ol.2019.10371.

27. Xue, J.M.; Liu, Y.; Wan, L.H.; Zhu, Y.X. Comprehensive analysis of differential gene expression to identify common gene signatures in multiple cancers. *Med. Sci. Monit.* **2020**, *26*, doi:10.12659/MSM.919953.

28. Wang, M.; Li, L.; Liu, J.; Wang, J. A gene interaction network-based method to measure the common and heterogeneous mechanisms of gynecological cancer. *Mol. Med. Rep.* **2018**, *18*, doi:10.3892/mmr.2018.8961.

29. Yuan, Y.; Shi, X.; Li, B.; Peng, M.; Zhu, T.; Lv, G.; Liu, L.; Jin, H.; Li, L.; Qin, D. Integrated analysis of key microRNAs /TFs /mRNAs/ in HPV-positive cervical cancer based on microRNA sequencing and bioinformatics analysis. *Pathol. Res. Pract.* **2020**, *216*, doi:10.1016/j.prp.2020.152952.

30. Mousavi, S.Z.; Poortahmasebi, V.; Mokhtari-Azad, T.; Shahmahmoodi, S.; Farahmand, M.; Farzanehpour, M.; Jalilvand, S. The dysregulation of microarray gene expression in cervical cancer is associated with overexpression of a unique messenger rna signature. *Iran. J. Microbiol.* **2020**, *12*, doi:10.18502/ijm.v12i6.5039.

31. Zhang, X.; Wang, Y. Identification of hub genes and key pathways associated with the progression of gynecological cancer. *Oncol. Lett.* **2019**, *18*, doi:10.3892/ol.2019.11004.

32. He, Z.; Wang, X.; Yang, Z.; Jiang, Y.; Li, L.; Wang, X.; Song, Z.; Wang, X.; Wan, J.; Jiang, S.; et al. Expression and prognosis of CDC45 in cervical cancer based on the GEO database. *PeerJ* **2021**, *9*, doi:10.7717/peerj.12114.

33. Chen, Q.; Zeng, X.; Huang, D.; Qiu, X. Identification of differentially expressed miRNAs in early-stage cervical cancer with lymph node metastasis across the cancer genome atlas datasets. *Cancer Manag. Res.* **2018**, *10*, doi:10.2147/CMAR.S183488.

34. Li, S.; Han, F.; Qi, N.; Wen, L.; Li, J.; Feng, C.; Wang, Q. Determination of a six-gene prognostic model for cervical cancer based on WGCNA combined with LASSO and Cox-PH analysis. *World J. Surg. Oncol.* **2021**, *19*, 1–11, doi:10.1186/s12957-021-02384-2.

35. Fu, X.H.; Wu, Y.F.; Xue, F. Probing pathway-related modules in invasive squamous cervical cancer based on topological centrality of network strategy. *J. Cancer Res. Ther.* **2018**, *14*, doi:10.4103/0973-1482.187352.

36. Wu, B.; Xi, S. Bioinformatics analysis of the transcriptional expression of minichromosome maintenance proteins as potential indicators of survival in patients with cervical cancer. *BMC Cancer* **2021**, *21*, doi:10.1186/s12885-021-08674-y.

37. Meng, H.; Liu, J.; Qiu, J.; Nie, S.; Jiang, Y.; Wan, Y.; Cheng, W. Identification of Key Genes in Association with Progression and Prognosis in Cervical Squamous Cell Carcinoma. *DNA Cell Biol.* **2020**, *39*, doi:10.1089/dna.2019.5202.

38. Ding, H.; Zhang, L.; Zhang, C.; Song, J.; Jiang, Y. Screening of Significant Biomarkers Related to Prognosis of Cervical Cancer and Functional Study Based on lncRNA-associated ceRNA Regulatory Network. *Comb. Chem. High Throughput Screen.* **2020**, *24*, doi:10.2174/1386207323999200729113028.

39. Li, S.; Liu, N.; Piao, J.; Meng, F.; Li, Y. Ccnb1 expedites the progression of cervical squamous cell carcinoma via the regulation by foxm1. *Onco. Targets. Ther.* **2020**, *13*, doi:10.2147/OTT.S279951.

40. Wu, B.; Xi, S. Bioinformatics analysis of differentially expressed genes and pathways in the development of cervical cancer. *BMC Cancer* **2021**, *21*, doi:10.1186/s12885-021-08412-4.

41. Wen, X.; Liu, S.; Cui, M. Effect of BRCA1 on the Concurrent Chemoradiotherapy Resistance of Cervical Squamous Cell Carcinoma Based on Transcriptome Sequencing Analysis. *Biomed Res. Int.* **2020**, *2020*, doi:10.1155/2020/3598417.

42. Suman, S.; Mishra, A. Network analysis revealed aurora kinase dysregulation in five gynecological types of cancer. *Oncol. Lett.* **2018**, *15*, doi:10.3892/ol.2017.7368.

43. Zhang, X.; Bai, J.; Yuan, C.; Long, L.; Zheng, Z.; Wang, Q.; Chen, F.; Zhou, Y. Bioinformatics analysis and identification of potential genes related to pathogenesis of cervical intraepithelial neoplasia. *J. Cancer* **2020**, *11*, doi:10.7150/jca.38211.

44. Sun, D.; Han, L.; Cao, R.; Wang, H.; Jiang, J.; Deng, Y.; Yu, X. Prediction of a miRNA-mRNA functional synergistic network for cervical squamous cell carcinoma. *FEBS Open Bio* **2019**, *9*, doi:10.1002/2211-5463.12747.

45. Oany, A.R.; Mia, M.; Pervin, T.; Alyami, S.A.; Moni, M.A. Integrative systems biology approaches to identify potential biomarkers and pathways of cervical cancer. *J. Pers. Med.* **2021**, *11*, doi:10.3390/jpm11050363.

46. Xiao, L.; Zhang, S.; Zheng, Q.; Zhang, S. Dysregulation of KIF14 regulates the cell cycle and predicts poor prognosis in cervical cancer: A study based on integrated approaches. *Brazilian J. Med. Biol. Res.* **2021**, *54*, 1–10, doi:10.1590/1414-431X2021e11363.

47. Yu, D.; Li, Y.; Ming, Z.; Wang, H.; Dong, Z.; Qiu, L.; Wang, T. Comprehensive circular RNA expression profile in radiation-treated HeLa cells and analysis of radioresistance-related circRNAs. *PeerJ* **2018**, *2018*, doi:10.7717/peerj.5011.

48. Zhang, X.; Yang, P.; Luo, X.; Su, C.; Chen, Y.; Zhao, L.; Wei, L.; Zeng, H.; Varghese, Z.; Moorhead, J.F.; et al. High olive oil diets enhance cervical tumour growth in mice: Transcriptome analysis for potential candidate genes and pathways. *Lipids Health Dis.* **2019**, *18*, doi:10.1186/s12944-019-1023-6.

49. Xu, F.; Shen, J.; Xu, S. Multi-Omics Data Analyses Construct a Six Immune-Related Genes Prognostic Model for Cervical Cancer in Tumor Microenvironment. *Front. Genet.* **2021**, *12*, doi:10.3389/fgene.2021.663617.

50. Jiang, P.; Cao, Y.; Gao, F.; Sun, W.; Liu, J.; Ma, Z.; Xie, M.; Fu, S. SNX10 and PTGDS are associated with the progression and prognosis of cervical squamous cell carcinoma. *BMC Cancer* **2021**, *21*, doi:10.1186/s12885-021-08212-w.

51. Yang, C.; Xu, X.; Jin, H. Identification of potential miRNAs and candidate genes of cervical intraepithelial neoplasia by bioinformatic analysis. *Eur. J. Gynaecol. Oncol.* **2016**, *37*, doi:10.12892/ejgo3131.2016.

52. Luo, H.; Li, Y.; Zhao, Y.; Chang, J.; Zhang, X.; Zou, B.; Gao, L.; Wang, W. Comprehensive Analysis of circRNA Expression Profiles During Cervical Carcinogenesis. *Front. Oncol.* **2021**, *11*, 1–13, doi:10.3389/fonc.2021.676609.

53. Tong, Y.; Sun, P.; Yong, J.; Zhang, H.; Huang, Y.; Guo, Y.; Yu, J.; Zhou, S.; Wang, Y.; Wang, Y.; et al. Radiogenomic Analysis of Papillary Thyroid Carcinoma for Prediction of Cervical Lymph Node Metastasis: A Preliminary Study. *Front. Oncol.* **2021**, *11*, doi:10.3389/fonc.2021.682998.

54. Zhang, Z.; Zhao, S.; Wang, K.; Shang, M.; Chen, Z.; Yang, H.; Chen, Y.; Chen, B. Identification of biomarkers associated with cervical lymph node metastasis in papillary thyroid carcinoma: Evidence from an integrated bioinformatic analysis. *Clin. Hemorheol. Microcirc.* **2021**, *78*, doi:10.3233/CH-201074.

55. Marquina, G.; Manzano, A.; Casado, A. Targeted Agents in Cervical Cancer: Beyond Bevacizumab. *Curr. Oncol. Rep.* **2018**, *20*, doi:10.1007/s11912-018-0680-3.

56. Sharma, S.; Deep, A.; Sharma, A.K. Current Treatment for Cervical Cancer: An Update. *Anticancer. Agents Med. Chem.* **2020**, *20*, doi:10.2174/1871520620666200224093301.

57. Duenas-Gonzalez, A.; Gonzalez-Fierro, A. Pharmacodynamics of current and emerging treatments for cervical cancer. *Expert Opin. Drug Metab. Toxicol.* **2019**, *15*, doi:10.1080/17425255.2019.1648431.

58. Barra, F.; Lorusso, D.; Leone Roberti Maggiore, U.; Ditto, A.; Bogani, G.; Raspagliesi, F.; Ferrero, S. Investigational drugs for the treatment of cervical cancer. *Expert Opin. Investig. Drugs* 2017, *26*.

59. Serrano-Olvera, A.; Cetina, L.; Coronel, J.; Dueñas-González, A. Emerging drugs for the treatment of cervical cancer. *Expert Opin. Emerg. Drugs* 2015, *20*.

60. Kamura, T.; Ushijima, K. Chemotherapy for advanced or recurrent cervical cancer. *Taiwan. J. Obstet. Gynecol.* 2013, *52*.

61. Klopp, A.H.; Eifel, P.J. Chemoradiotherapy for cervical cancer in 2010. *Curr. Oncol. Rep.* **2011**, *13*, doi:10.1007/s11912-010-0134-z.

62. Tao, X.; Hu, W.; Ramirez, P.T.; Kavanagh, J.J. Chemotherapy for recurrent and metastatic cervical cancer. *Gynecol. Oncol.* **2008**, *110*, doi:10.1016/j.ygyno.2008.04.024.

63. Diaz-Padilla, I.; Monk, B.J.; Mackay, H.J.; Oaknin, A. Treatment of metastatic cervical cancer: Future directions involving targeted agents. *Crit. Rev. Oncol. Hematol.* 2013, *85*.

64. Tierney, J.F.; Vale, C.; Symonds, P. Concomitant and Neoadjuvant Chemotherapy for Cervical Cancer. *Clin. Oncol.* **2008**, *20*, doi:10.1016/j.clon.2008.04.003.

65. Verschraegen, C.F. Irinotecan for the treatment of cervical cancer. *Oncology (Williston Park).* 2002, *16*.

66. Su, J.; Zhang, F.; Li, X.; Liu, Z. Osthole promotes the suppressive effects of cisplatin on NRF2 expression to prevent drug-resistant cervical cancer progression. *Biochem. Biophys. Res. Commun.* **2019**, *514*, doi:10.1016/j.bbrc.2019.04.021.

67. Ackermann, S.; Beckmann, M.W.; Thiel, F.; Bogenrieder, T. Topotecan in cervical cancer. *Int. J. Gynecol. Cancer* 2007, *17*.

68. Chandimali, N.; Sun, H.N.; Park, Y.H.; Kwon, T. BRM270 suppresses cervical cancer stem cell characteristics and progression by inhibiting SOX2. *In Vivo (Brooklyn).* **2020**, *34*, doi:10.21873/invivo.11879.

69. Markman, M. Advances in cervical cancer pharmacotherapies. *Expert Rev. Clin. Pharmacol.* 2014, *7*.

70. Moga, M.A.; Dima, L.; Balan, A.; Blidaru, A.; Dimienescu, O.G.; Podasca, C.; Toma, S. Are bioactive molecules from seaweeds a novel and challenging option for the prevention of HPV infection and cervical cancer therapy?—a review. *Int. J. Mol. Sci.* 2021, *22*.

71. Lee, S.W.; Kim, Y.M.; Kim, M.B.; Kim, D.Y.; Kim, J.H.; Nam, J.H.; Kim, Y.T. Chemosensitivity of uterine cervical cancer demonstrated by the histoculture drug response assay. *Tohoku J. Exp. Med.* **2009**, *219*, doi:10.1620/tjem.219.277.

72. Small, W. Potential for use of amifostine in cervical cancer. *Semin. Oncol.* **2002**, *29*, doi:10.1053/sonc.2002.37366.

73. Ai, Z.; Wang, J.; Xu, Y.; Teng, Y. Bioinformatics analysis reveals potential candidate drugs for cervical cancer. *J. Obstet. Gynaecol. Res.* **2013**, *39*, doi:10.1111/jog.12022.

74. Ujhelyi, Z.; Kalantari, A.; Vecsernyés, M.; Róka, E.; Fenyvesi, F.; Póka, R.; Kozma, B.; Bácskay, I. The enhanced inhibitory effect of different antitumor agents in self-microemulsifying drug delivery systems on human cervical cancer HeLa cells. *Molecules* **2015**, *20*, doi:10.3390/molecules200713226.
